# Supplementary material for: Maternal immunisation against Group B Streptococcus: A global analysis of health impact and cost-effectiveness
Source: PLoS Med. 2023 Mar 14;20(3):e1004068. doi: 10.1371/journal.pmed.1004068 (PMC10013922; doi:10.1371/journal.pmed.1004068)
Supplement: S1 Appendix — Table A. Updated Consolidated Health Economic Evaluation Reporting Standards (CHEERS) checklist from [53]. Table B. Model parameter values used in base case analysis. (a) For parameters estimated in our previously published burden model, we used samples of the posterior distribution of the parameter estimates, and these samples along with other model input data are provided online. Values presented in this table correspond to the mean of the posterior distributions. (b) Regions for these parameters are based on a country’s World Bank income classification. (c) See section A2.7. (d) See section A2.6. (e) For these parameters, countries are assigned to the “developed” region according to the World Bank development status; for countries not classified as “developed,” the region is based on the UN geographical region. (f) Regions for these parameters are based on a country’s World Bank development status. (g) In the base case, we conservatively assume no excess risk of mild NDI following GBS by setting the value for these parameters to the match the baseline risk of mild NDI, i.e., we assume Riskmild-NDI-sep = Riskmild-NDI-men = Riskmild-NDI-baseline. EOGBS, early-onset GBS; GBS, Group B Streptococcus; HIC, high-income country; iGBS, invasive GBS; LIC, low-income country; LMIC, lower middle-income country; LOGBS, late-onset GBS; NDI, neurodevelopmental impairment; OR, odds ratio; UMIC, upper middle-income country. Table C. Country-specific model inputs values. (a) Estimated using regression model (see section A2.6 for further details). (b) Estimated using regression model (see section A2.7 for further details). (c) Values in italics were based on the average across other high-income countries. (d) See section A2.5 for further details on how these values were estimated. (e) Based on estimates World Bank where available, otherwise based on estimated from the IMF. (f) Based on values from Ochalek and colleagues and Woods and colleagues—see section A2.8 for how these values [file pmed.1004068.s001.docx]

**Supplementary appendix to:** Maternal immunisation against Group B Streptococcus: a global analysis of health impact and cost-effectiveness

**Authors**

Simon R. Procter^1,2*^, Bronner P. Gonçalves^1,2^, Proma Paul^1,2^, Jaya Chandna^1,2^, Farah Seedat^1,2^, Artemis Koukounari^1,2^, Raymond Hutubessy^3^, Caroline Trotter^4^, Joy E Lawn^1,2^, Mark Jit^1,5*^

*Corresponding authors: simon.procter@lshtm.ac.uk; mark.jit@lshtm.ac.uk

**Affiliations**

1. Department of Infectious Disease Epidemiology, London School of Hygiene & Tropical Medicine, London, United Kingdom

2. Maternal, Adolescent, Reproductive & Child Health (MARCH) Centre, London School of Hygiene & Tropical Medicine, London, United Kingdom

3. Department of Immunization, Vaccines and Biologicals (IVB), World Health Organization, Geneva, Switzerland

4. Disease Dynamics Unit, Department of Veterinary Medicine, University of Cambridge, Cambridge, United Kingdom

5. School of Public Health, University of Hong Kong, Hong Kong SAR, China

## Contents

[Contents 2](#_Toc104298733)

[A1 CHEERS Checklist 3](#_Toc104298734)

[A2 Model Inputs 5](#_Toc104298735)

[A2.1 Base case parameters 5](#_Toc104298736)

[A2.2 Scenario Analysis 13](#_Toc104298737)

[A2.3 Calculation of GBS-stillbirth risk 13](#_Toc104298738)

[A2.4 Calculation of GBS-associated preterm risk 13](#_Toc104298739)

[A2.5 Proportion of GBS-associated preterm births protected by vaccination 14](#_Toc104298740)

[A2.6 Estimation of acute costs 17](#_Toc104298741)

[A2.7 Estimation of vaccine delivery costs 19](#_Toc104298742)

[A2.8 Cost-effectiveness thresholds 20](#_Toc104298743)

[A3 Supplementary Results 21](#_Toc104298744)

[A4 References 28](#_Toc104298745)

## A1 CHEERS Checklist

**Table A: Updated Consolidated Health Economic Evaluation Reporting Standards (CHEERS) checklist from reference [1].**

| **Topic** | **No.** | **Item** | **Location where item is reported** |
| --- | --- | --- | --- |
| **Title and abstract** | | | |
| Title | 1 | Identify the study as an economic evaluation and specify the interventions being compared. | See title is on page 1 |
| Abstract | 2 | Provide a structured summary that highlights context, key methods, results, and alternative analyses. | See abstract on page 2 |
| **Introduction** | | | |
| Background and objectives | 3 | Give the context for the study, the study question, and its practical relevance for decision making in policy or practice. | See introduction on pages 4 and 5 |
| **Methods** | | | |
| Health economic analysis plan | 4 | Indicate whether a health economic analysis plan was developed and where available. | Not applicable as not RCT based |
| Study population | 5 | Describe characteristics of the study population (such as age range, demographics, socioeconomic, or clinical characteristics). | Methods para 1 on page 5 |
| Setting and location | 6 | Provide relevant contextual information that may influence findings. | Methods para 1 on page 5 |
| Comparators | 7 | Describe the interventions or strategies being compared and why chosen. | Methods para 3 on page 6 |
| Perspective | 8 | State the perspective(s) adopted by the study and why chosen. | Methods para 11 on page 8 |
| Time horizon | 9 | State the time horizon for the study and why appropriate. | Methods para 3 on page 6 |
| Discount rate | 10 | Report the discount rate(s) and reason chosen. | Methods para 14 on page 9 |
| Selection of outcomes | 11 | Describe what outcomes were used as the measure(s) of benefit(s) and harm(s). | Methods para 8 on pages 7-8 |
| Measurement of outcomes | 12 | Describe how outcomes used to capture benefit(s) and harm(s) were measured. | Methods para 8 on pages 7-8 |
| Valuation of outcomes | 13 | Describe the population and methods used to measure and value outcomes. | Methods para 8 on pages 7-8 |
| Measurement and valuation of resources and costs | 14 | Describe how costs were valued. | Methods para 11 and 12 on page 9, and S1 Appendix A2.6 and A2.7 |
| Currency, price date, and conversion | 15 | Report the dates of the estimated resource quantities and unit costs, plus the currency and year of conversion. | Methods para 11 and 12 on page 9, and S1 Appendix A2.6 and A2.7 |
| Rationale and description of model | 16 | If modelling is used, describe in detail and why used. Report if the model is publicly available and where it can be accessed. | Methods para 2 and Fig 1 on pages 5-6, data sharing statement on page 19: code and data available online |
| Analytics and assumptions | 17 | Describe any methods for analysing or statistically transforming data, any extrapolation methods, and approaches for validating any model used. | Methods and S1 Appendix |
| Characterising heterogeneity | 18 | Describe any methods used for estimating how the results of the study vary for subgroups. | Not applicable |
| Characterising distributional effects | 19 | Describe how impacts are distributed across different individuals or adjustments made to reflect priority populations. | Not applicable |
| Characterising uncertainty | 20 | Describe methods to characterise any sources of uncertainty in the analysis. | Methods para 10 on page 8 and para 17 on page 10, and S1 Appendix A2.1 and A2.2 |
| Approach to engagement with patients and others affected by the study | 21 | Describe any approaches to engage patients or service recipients, the general public, communities, or stakeholders (such as clinicians or payers) in the design of the study. | Not applicable |
| **Results** | | | |
| Study parameters | 22 | Report all analytic inputs (such as values, ranges, references) including uncertainty or distributional assumptions. | S1 Appendix tables 1 and 2 |
| Summary of main results | 23 | Report the mean values for the main categories of costs and outcomes of interest and summarise them in the most appropriate overall measure. | Results paras 1 to 4, table 2, and Fig 2 on pages 11 to 13 |
| Effect of uncertainty | 24 | Describe how uncertainty about analytic judgments, inputs, or projections affect findings. Report the effect of choice of discount rate and time horizon, if applicable. | Results para 7 on pages 14-15, Figs. 2 and 4, and S1 Appendix Figs. 1 to 4 |
| Effect of engagement with patients and others affected by the study | 25 | Report on any difference patient/service recipient, general public, community, or stakeholder involvement made to the approach or findings of the study | Not applicable, no patients involved in the study |
| **Discussion** | | | |
| Study findings, limitations, generalisability, and current knowledge | 26 | Report key findings, limitations, ethical or equity considerations not captured, and how these could affect patients, policy, or practice. | Discussion on pages 16 to 18 |
| **Other relevant information** | | | |
| Source of funding | 27 | Describe how the study was funded and any role of the funder in the identification, design, conduct, and reporting of the analysis | Methods final para page 10 and acknowledgements on page 18 |
| Conflicts of interest | 28 | Report authors conflicts of interest according to journal or International Committee of Medical Journal Editors requirements. | Declaration of interests on page 19 |

## A2 Model Inputs

This section provides additional information on model input data and parameterisation that have been described in the methods section of the main paper. Data input files and R code used to conduct the simulations and analysis are available in the project GitHub repository at https://github.com/mert0248/GBS-vax-econ-model

### A2.1 Base case parameters

**Table B: Model parameter values used in base case analysis.**

a. For parameters estimated in our previously published burden model we used samples of the posterior distribution of the parameter estimates, and these samples along with other model input data are provided online. Values presented in this table correspond to the mean of the posterior distributions.

b. Regions for these parameters are based on a country’s World Bank income classification.

c. See section A2.7.

d. See section A2.6.

e. For these parameters countries are assigned to the “developed” region according to the World Bank development status; for countries not classified as “developed” the region is based on the UN geographical region.

f. Regions for these parameters are based on a country’s World Bank development status.

g. In the base case we conservatively assume no excess risk of mild NDI following GBS by setting the value for these parameters to the match the baseline risk of mild NDI, i.e., we assume *Risk_mild-NDI-sep_* = *Risk_mild-NDI-men_* = *Risk_mild-NDI-baseline_*.

GBS = Group B Streptococcus; iGBS = invasive GBS; EOGBS = Early-Onset GBS; LOGBS = Late-Onset GBS; NDI = Neurodevelopmental Impairment; LIC = Low-Income Country; LMIC = Lower Middle-Income Country; UMIC = Upper Middle-Income Country; HIC = High-Income Country; OR = Odds Ratio.

| **Parameter** | **Description** | **Region** | **Value** | **Distribution** | **Reference** |
| --- | --- | --- | --- | --- | --- |
|  | | | | | |
| *VE*_iGBS_ | Vaccine efficacy (iGBS disease) | Global | 80% | Fixed | Assumption based on [2] |
| *VE_GBS-stillbirth_* | Vaccine efficacy (GBS stillbirth) | Global | 80% | Fixed | Assumption based on [2] |
| *VE_GBS-preterm_* | Vaccine efficacy (GB-associated preterm) | Global | 0% | Fixed | Assumption based on [2] |
| *N_doses_* | Number of vaccine doses per course | Global | 1 | Fixed | Assumption based on [2] |
| *V_coverage_* | Vaccination coverage | Country-specific based on ANC4 coverage | | Fixed | [3] |
|  | | | | | |
| *Cost_purchase_* | Vaccine purchase price (per dose)^b^ | LIC | $3.5 | Fixed | [4] |
|  |  | LMIC | $3.5 | Fixed |  |
|  |  | UMIC | $15 | Fixed |  |
|  |  | HIC | $50 | Fixed |  |
| *Cost_delivery_* | Cost of vaccine delivery^c^ | Country-specific from regression | | Lognormal | [5] |
| *Cost_acute_* | Cost of acute care episode^d^ | Country-specific from regression | | Lognormal | [6–8] |
| *Cost_disability_* | Annual cost of long-term disability (mod/sev NDI) as a proportion of acute care costs | Global | 0.16 | Uniform (0.04, 0.28) | Assumption based on [9,10] |
|  | | | | | |
| *U_term-birth_* | Utility decrement for term birth | Global | 0 | Fixed | Assumption |
| *U_preterm-birth_* | Utility decrement for preterm birth | Global | 0.066 | Beta (15.89, 224.9) | [11] |
| *U_mild-NDI_* | Utility decrement for mild NDI | Global | 0.179 | Beta (14.91, 68.40) | [9] |
| *U_moderate-NDI_* | Utility decrement for moderate NDI | Global | 0.298 | Beta (20.61, 48.55) | [9] |
| *U_severe-NDI_* | Utility decrement for severe NDI | Global | 0.595 | Beta (19.50, 15.45) | [9] |
| *U_acute-meningitis_* | Utility decrement for acute meningitis | Global | 0.0232 | Beta (0.082, 3.46) | [12] |
| *U_acute-sepsis_* | Utility decrement for acute sepsis | Global | 0.0079 | Beta (0.069, 8.64) | [12] |
| *LOS* | Length of acute episode in days | Global | 29 | Fixed | [6] |
|  | | | | | |
| *Risk_mat-col_* | Risk of maternal colonisation with GBS | Country-specific | | Posterior sample^a^ | [13] |
| *Risk_EOGBS-col_* | Risk of EOGBS if born to colonised mothers | Country-specific | | Posterior sample^a^ | [13] |
| *Risk_EOGBS-no-col_* | Risk of EOGBS if born to non-colonised mothers | Global | 0 | Fixed |  |
| *Prop_LOGBS_* | Proportion of iGBS that is LOGBS^e^ | Developed | 0.376 | Posterior sample^a^ | [13] |
|  |  | Africa | 0.467 |  |  |
|  |  | Asia | 0.335 |  |  |
|  |  | Latin America and the Caribbean | 0.407 |  |  |
|  |  | Oceania | 0.410 |  |  |
| *CFR_EOGBS_* | Case-fatality risk for EOGBS^e^ | Developed | 0.065 | Posterior sample^a^ | [13] |
|  |  | Africa | 0.235 |  |  |
|  |  | Asia | 0.153 |  |  |
|  |  | Latin America and the Caribbean | 0.162 |  |  |
|  |  | Oceania | 0.188 |  |  |
| *CFR_LOGBS_* | Case-fatality risk for LOGBS^e^ | Developed | 0.061 | Posterior sample^a^ | [13] |
|  |  | Africa | 0.108 |  |  |
|  |  | Asia | 0.069 |  |  |
|  |  | Latin America and the Caribbean | 0.088 |  |  |
|  |  | Oceania | 0.106 |  |  |
| *Prop_men-EOGBS_* | Proportion of EOGBS that is meningitis | Global | 0.130 | Posterior sample^a^ | [13] |
| *Prop_men-LOGBS_* | Proportion of LOGBS that is meningitis | Global | 0.407 | Posterior sample^a^ | [13] |
| *Risk_mild-NDI-men_* | Risk of mild NDI after GBS meningitis^g^ | Global | 0.0261 | Fixed | [14] |
| *Risk_mod_sev-NDI-men_* | Risk of moderate/severe NDI after GBS meningitis | Global | 0.207 | Posterior sample^a^ | [13] |
| *Risk_mild-NDI-sep_* | Risk of mild NDI after GBS sepsis^g^ | Global | 0.0261 | Fixed | [14] |
| *Risk_mod_sev-NDI-sep_* | Risk of moderate/severe NDI after GBS sepsis^f^ | Developed | 0.033 | Posterior sample^a^ | [13] |
|  |  | Developing | 0.092 | Posterior sample^a^ | [13] |
| *Prop_severe_* | Proportion of mod/severe NDI that is severe | Global | 0.330 | Fixed | [14] |
| *Risk_mild-NDI-baseline_* | Baseline risk of mild NDI without GBS | Global | 0.0261 | Fixed | [14] |
| *Risk_mod_sev-NDI-baseline_* | Baseline risk of moderate/severe NDI without GBS | Global | 0.0214 | Fixed | [14] |
| *Prop_stillbirth-GBS_* | Proportion of stillbirths due to GBS^e^ | Developed | 0.022 | Posterior sample^a^ | [13] |
|  |  | Africa | 0.026 |  |  |
|  |  | Asia | 0.027 |  |  |
|  |  | Latin America and the Caribbean | 0.032 |  |  |
|  |  | Oceania | 0.032 |  |  |
| *OR_preterm-col_* | OR for preterm births amongst colonised mothers | Global | 1.321 | Posterior sample^a^ | [13] |
|  | | | | | |

**Table C: Country-specific model inputs values**

1. Estimated using regression model (see section A2.6 for further details)
2. Estimated using regression model (see section A2.7 for further details)
3. Values in italics were based on the average across other high-income countries
4. See section A2.5 for further details on how these values were estimated
5. Based on estimates World Bank where available, otherwise based on estimated from the IMF.
6. Based on values from Ochalek et al. and Woods et al. - see section A2.8 for how these values were calculated; values in italics were imputed using regression against GDP per capita

ANC = Antenatal Care; CET = Cost-Effectiveness Threshold; EOGBS = Early-Onset Group B Streptococcus Disease; GDP = Gross Domestic Product; USD = United States Dollars.

| **Country ISO3 code** | **Total births**  **[15]** | **Stillbirth risk**  **[3]** | **Preterm risk**  **[16]** | **Maternal colonisation risk** | **EOGBS risk in births to colonised mothers** | **Cost of acute episode (2020 USD)^a^** | **Cost of vaccine delivery (2020 USD)^b^** | **Life expectancy at birth**  **[15]** | **Coverage SBA^c^**  **[17]** | **ANC1 coverage**  **[3]** | **ANC4 coverage**  **[3]** | **Proportion of preterm births protected among effectively vaccinated mothers^d^** | **GDP per capita (2020 USD)^e^**  **[18,19]** | **Empirical CET (2020 USD)^f^**  **[20,21]** |
| --- | --- | --- | --- | --- | --- | --- | --- | --- | --- | --- | --- | --- | --- | --- |
| AFG | 1204853 | 0.0284 | 0.104 | 0.258 | 0.0086 | 123.99 | 0.32 | 64.8 | 58.8% | 69.2% | 24.0% | 0.586 | 586 | 171.04 |
| AGO | 1243046 | 0.0198 | 0.120 | 0.148 | 0.0161 | 136.58 | 0.90 | 61.1 | 49.6% | 90.3% | 70.5% | 0.557 | 2974 | 745.99 |
| ALB | 34015 | 0.0041 | 0.087 | 0.123 | 0.0013 | 697.71 | 2.26 | 78.6 | 99.8% | 91.7% | 81.5% | 0.784 | 5323 | 3816.68 |
| ARE | 99413 | 0.0049 | 0.055 | 0.189 | 0.0161 | 7209.49 | 12.37 | 78.0 | 99.2% | 99.5% | 87.1% | 0.852 | 39180 | *24638.59* |
| ARG | 755237 | 0.0053 | 0.084 | 0.070 | 0.0025 | 3165.28 | 3.46 | 76.7 | 99.5% | 99.2% | 95.5% | 0.852 | 9890 | 4139.38 |
| ARM | 41700 | 0.0129 | 0.104 | 0.098 | 0.0161 | 1532.94 | 1.89 | 75.1 | 99.8% | 99.2% | 96.4% | 0.716 | 4605 | 1514.44 |
| ATG | 1480 | 0.0055 | 0.098 | 0.134 | 0.0161 | 2419.37 | 5.32 | 77.0 | 100.0% | 99.1% | 98.1% | 0.852 | 17195 | 5904.94 |
| AUS | 318314 | 0.0022 | 0.086 | 0.230 | 0.0013 | 27656.98 | 16.99 | 82.9 | 98.7% | 98.4% | 92.3% | 0.852 | 54464 | 33962.2 |
| AUT | 87850 | 0.0022 | 0.079 | 0.173 | 0.0013 | 26481.33 | 16.07 | 81.8 | 98.4% | 99.5% | 99.0% | 0.852 | 50247 | 29978.06 |
| AZE | 168806 | 0.009 | 0.104 | 0.146 | 0.0161 | 454.34 | 1.87 | 73.0 | 99.4% | 90.0% | 68.4% | 0.604 | 4814 | 1287.53 |
| BDI | 432821 | 0.0261 | 0.120 | 0.118 | 0.0161 | 30.95 | 0.17 | 61.6 | 85.1% | 99.4% | 41.9% | 0.479 | 257 | 96.79 |
| BEL | 124375 | 0.0028 | 0.101 | 0.189 | 0.0013 | 24713.97 | 15.05 | 81.7 | *99.0%* | 99.5% | 99.1% | 0.852 | 46542 | 26734.34 |
| BEN | 413132 | 0.0203 | 0.093 | 0.151 | 0.0161 | 46.8 | 0.68 | 61.8 | 78.1% | 90.3% | 54.0% | 0.615 | 1218 | 332.44 |
| BFA | 744929 | 0.0195 | 0.120 | 0.146 | 0.0161 | 73.11 | 0.49 | 61.6 | 79.8% | 96.2% | 58.3% | 0.52 | 775 | 223.06 |
| BGD | 2946484 | 0.0243 | 0.191 | 0.100 | 0.0086 | 80.44 | 0.97 | 72.6 | 59.0% | 86.3% | 41.5% | 0.553 | 1855 | 225.59 |
| BGR | 63355 | 0.005 | 0.090 | 0.163 | 0.0025 | 2178.05 | 3.99 | 74.9 | 99.8% | 98.6% | 94.3% | 0.548 | 9863 | 7609.8 |
| BHR | 21806 | 0.0059 | 0.103 | 0.228 | 0.0161 | 3142.27 | 7.41 | 77.3 | 99.9% | 99.4% | 98.3% | 0.852 | 25930 | 12327.61 |
| BHS | 5386 | 0.0116 | 0.098 | 0.166 | 0.0161 | 8002.45 | 8.94 | 73.9 | 99.0% | 96.3% | 86.8% | 0.852 | 35664 | *15797.7* |
| BIH | 27490 | 0.0028 | 0.067 | 0.141 | 0.0013 | 1640.41 | 2.57 | 77.4 | 99.9% | 99.1% | 96.9% | 0.549 | 6120 | 1969.84 |
| BLR | 111818 | 0.002 | 0.043 | 0.138 | 0.0013 | 1099.95 | 2.69 | 74.2 | 99.9% | 99.4% | 99.3% | 0.544 | 6798 | 4593.27 |
| BLZ | 7922 | 0.0065 | 0.098 | 0.190 | 0.0161 | 755.26 | 1.83 | 74.6 | 94.0% | 98.0% | 89.3% | 0.557 | 4699 | 3384.74 |
| BOL | 246637 | 0.0089 | 0.098 | 0.122 | 0.0161 | 609.3 | 1.45 | 71.5 | 81.1% | 92.7% | 79.0% | 0.729 | 3566 | 2655.3 |
| BRA | 2934460 | 0.0075 | 0.112 | 0.182 | 0.0025 | 2788.53 | 2.83 | 75.9 | 99.1% | 96.4% | 92.0% | 0.684 | 8932 | 6321.06 |
| BRB | 3062 | 0.0074 | 0.098 | 0.229 | 0.0161 | 3997.32 | 5.77 | 79.2 | 99.1% | 98.4% | 84.9% | 0.852 | 18139 | *8655.68* |
| BRN | 6391 | 0.0046 | 0.104 | 0.123 | 0.0161 | 2077.62 | 9.64 | 75.9 | 99.8% | 98.5% | 98.5% | 0.852 | 29314 | 24369.34 |
| BTN | 13115 | 0.0097 | 0.104 | 0.171 | 0.0161 | 245.22 | 1.40 | 71.8 | 96.3% | 98.1% | 84.2% | 0.532 | 3371 | 831.22 |
| BWA | 56157 | 0.0152 | 0.120 | 0.355 | 0.0161 | 1382.33 | 2.69 | 69.6 | 99.8% | 98.0% | 90.6% | 0.572 | 7979 | 2722.08 |
| CAF | 165064 | 0.0298 | 0.120 | 0.204 | 0.0161 | 62.64 | 0.31 | 53.3 | 40.3% | 81.1% | 43.0% | 0.592 | 480 | 113.84 |
| CAN | 385533 | 0.0028 | 0.082 | 0.201 | 0.0013 | 25263.04 | 14.49 | 82.0 | 98.0% | 99.1% | 98.2% | 0.852 | 46400 | 28401.29 |
| CHE | 87470 | 0.0022 | 0.072 | 0.194 | 0.0013 | 57038.59 | 27.21 | 83.7 | *99.0%* | 99.6% | 99.3% | 0.852 | 85686 | 50342.09 |
| CHL | 231145 | 0.0031 | 0.079 | 0.134 | 0.0025 | 5023.04 | 5.06 | 80.2 | 99.8% | 95.1% | 93.2% | 0.852 | 14616 | 6986.34 |
| CHN | 16977981 | 0.0055 | 0.069 | 0.098 | 0.0161 | 1572.89 | 4.11 | 76.9 | 99.9% | 97.4% | 82.8% | 0.562 | 10243 | 6260.87 |
| CIV | 890284 | 0.0232 | 0.120 | 0.191 | 0.0161 | 145.28 | 1.13 | 57.8 | 73.6% | 94.8% | 54.3% | 0.56 | 2228 | 383.74 |
| CMR | 887451 | 0.0194 | 0.120 | 0.173 | 0.0161 | 97.81 | 0.79 | 59.3 | 69.0% | 91.1% | 69.9% | 0.543 | 1502 | 153.71 |
| COD | 3433710 | 0.0272 | 0.098 | 0.157 | 0.0161 | 30.95 | 0.33 | 60.7 | 85.2% | 94.0% | 57.5% | 0.537 | 574 | 72.07 |
| COG | 171433 | 0.015 | 0.120 | 0.168 | 0.0161 | 86.14 | 0.92 | 64.6 | 91.2% | 96.5% | 86.1% | 0.516 | 2746 | 1435.37 |
| COL | 739473 | 0.0071 | 0.145 | 0.104 | 0.0161 | 1430.94 | 2.29 | 77.3 | 98.8% | 97.9% | 92.2% | 0.749 | 6419 | 8281.9 |
| COM | 26423 | 0.0246 | 0.120 | 0.102 | 0.0161 | 138.86 | 0.74 | 64.3 | 82.2% | 96.4% | 67.1% | 0.606 | 1362 | 539.85 |
| CPV | 10639 | 0.0109 | 0.120 | 0.149 | 0.0161 | 411.31 | 1.42 | 73.0 | 97.3% | 99.6% | 86.3% | 0.557 | 3602 | 2451.42 |
| CRI | 70147 | 0.0045 | 0.098 | 0.192 | 0.0086 | 3065.11 | 4.69 | 80.3 | 98.5% | 98.7% | 94.8% | 0.557 | 12599 | 18545.15 |
| CUB | 115717 | 0.0069 | 0.060 | 0.187 | 0.0161 | 3522.92 | 3.78 | 78.8 | 100.0% | 99.3% | 98.6% | 0.556 | 8822 | *4802.83* |
| CYP | 12551 | 0.0025 | 0.187 | 0.140 | 0.0161 | 7962.09 | 9.67 | 81.0 | 99.3% | 99.3% | 98.8% | 0.852 | 28488 | 14975.15 |
| CZE | 111358 | 0.0026 | 0.076 | 0.193 | 0.0013 | 7216.9 | 8.22 | 79.1 | 99.8% | 99.3% | 97.8% | 0.852 | 23539 | 11511.5 |
| DEU | 779236 | 0.0027 | 0.089 | 0.177 | 0.0025 | 27738.53 | 15.36 | 80.9 | 98.8% | 99.1% | 98.9% | 0.852 | 46473 | 27956.1 |
| DJI | 20632 | 0.0279 | 0.120 | 0.226 | 0.0161 | 115.01 | 1.57 | 67.1 | 87.4% | 97.1% | 22.9% | 0.552 | 3103 | 599.46 |
| DNK | 61283 | 0.002 | 0.072 | 0.197 | 0.0025 | 31376.89 | 19.74 | 81.2 | 95.3% | 99.5% | 99.2% | 0.852 | 60300 | 37004.37 |
| DOM | 207971 | 0.0107 | 0.098 | 0.238 | 0.0161 | 1415.87 | 3.00 | 74.1 | 99.8% | 99.4% | 95.1% | 0.64 | 8596 | 4052.02 |
| DZA | 1031794 | 0.0095 | 0.134 | 0.207 | 0.0161 | 616.2 | 1.52 | 76.9 | 98.8% | 94.3% | 69.2% | 0.574 | 3940 | 4059.17 |
| ECU | 336114 | 0.0087 | 0.080 | 0.142 | 0.0161 | 1399.77 | 2.39 | 77.0 | 96.0% | 97.1% | 90.6% | 0.565 | 6261 | 5236.36 |
| EGY | 2584496 | 0.009 | 0.134 | 0.236 | 0.0161 | 334.16 | 1.62 | 72.0 | 91.5% | 95.2% | 89.6% | 0.711 | 3057 | 1222.45 |
| ERI | 105531 | 0.0183 | 0.120 | 0.107 | 0.0161 | 39.52 | 0.35 | 66.3 | 34.1% | 88.7% | 64.0% | 0.543 | 567 | 139.57 |
| ESP | 397799 | 0.0022 | 0.065 | 0.155 | 0.0013 | 11640.36 | 9.52 | 83.5 | *99.0%* | 99.4% | 98.8% | 0.852 | 29586 | 15155.39 |
| EST | 13678 | 0.0022 | 0.057 | 0.176 | 0.0013 | 6047.88 | 8.25 | 78.5 | 99.7% | 99.4% | 99.2% | 0.852 | 23720 | 11398.38 |
| ETH | 3514446 | 0.0246 | 0.120 | 0.134 | 0.0161 | 42.27 | 0.52 | 66.6 | 49.8% | 73.6% | 42.9% | 0.552 | 968 | 290.27 |
| FIN | 51530 | 0.002 | 0.058 | 0.173 | 0.0025 | 21581.14 | 16.11 | 81.8 | 100.0% | 99.7% | 99.3% | 0.852 | 48749 | 28223.35 |
| FJI | 18951 | 0.0086 | 0.100 | 0.094 | 0.0161 | 578.61 | 2.19 | 67.4 | 99.8% | 99.3% | 94.5% | 0.557 | 6144 | 1320.04 |
| FRA | 728763 | 0.0043 | 0.084 | 0.162 | 0.0013 | 21832.34 | 13.20 | 82.6 | 98.1% | 99.7% | 99.2% | 0.852 | 41811 | 22832.76 |
| FSM | 2559 | 0.0117 | 0.100 | 0.145 | 0.0161 | 1153.31 | 1.62 | 67.9 | 100.0% | 87.9% | 82.5% | 0.547 | 3952 | 1108.81 |
| GAB | 66765 | 0.0138 | 0.120 | 0.199 | 0.0161 | 517.61 | 2.86 | 66.5 | 89.3% | 98.0% | 83.7% | 0.593 | 8111 | 2305.38 |
| GBR | 771936 | 0.003 | 0.070 | 0.201 | 0.0025 | 20752.51 | 13.81 | 81.2 | *99.0%* | 99.2% | 98.7% | 0.852 | 42417 | 18271.33 |
| GEO | 54476 | 0.0057 | 0.104 | 0.132 | 0.0161 | 748.09 | 1.89 | 73.8 | 99.9% | 97.3% | 88.9% | 0.53 | 4694 | 1066.67 |
| GHA | 871269 | 0.0217 | 0.120 | 0.168 | 0.0161 | 145.72 | 1.08 | 64.1 | 78.9% | 98.5% | 88.9% | 0.611 | 2221 | 700.26 |
| GIN | 449023 | 0.0252 | 0.120 | 0.141 | 0.0161 | 74.56 | 0.64 | 61.6 | 55.3% | 90.1% | 62.6% | 0.575 | 1013 | 280.6 |
| GMB | 87245 | 0.0219 | 0.120 | 0.240 | 0.0161 | 47.95 | 0.44 | 62.1 | 83.8% | 99.5% | 80.1% | 0.531 | 774 | 469.6 |
| GNB | 65561 | 0.0322 | 0.120 | 0.193 | 0.0161 | 116.73 | 0.42 | 58.3 | 53.8% | 94.4% | 66.7% | 0.539 | 811 | 74.57 |
| GNQ | 43015 | 0.0151 | 0.120 | 0.242 | 0.0161 | 636.41 | 2.95 | 58.7 | 68.3% | 98.8% | 92.1% | 0.573 | 8106 | 6536.06 |
| GRC | 82635 | 0.0031 | 0.114 | 0.116 | 0.0013 | 5591.85 | 6.51 | 81.9 | 99.9% | 99.3% | 98.8% | 0.852 | 19147 | 9991.98 |
| GRD | 1843 | 0.0098 | 0.098 | 0.155 | 0.0025 | 1569.79 | 3.70 | 72.4 | 100.0% | 99.3% | 94.9% | 0.557 | 10735 | 3167.45 |
| GTM | 423040 | 0.0127 | 0.098 | 0.128 | 0.0161 | 685.72 | 2.02 | 74.3 | 69.8% | 96.6% | 83.9% | 0.649 | 4354 | 1783.79 |
| GUY | 15592 | 0.0138 | 0.098 | 0.111 | 0.0161 | 858.28 | 2.89 | 69.9 | 95.8% | 98.4% | 91.7% | 0.56 | 6594 | 3338.85 |
| HND | 207332 | 0.0085 | 0.098 | 0.140 | 0.0161 | 438.53 | 1.15 | 75.3 | 74.0% | 96.7% | 87.6% | 0.712 | 2551 | 2072.47 |
| HRV | 37234 | 0.003 | 0.050 | 0.160 | 0.0046 | 3557.31 | 5.36 | 78.4 | 100.0% | 99.4% | 98.7% | 0.852 | 14936 | 7137.75 |
| HTI | 271066 | 0.0199 | 0.098 | 0.154 | 0.0161 | 104.34 | 0.68 | 64.0 | 41.6% | 91.2% | 62.2% | 0.614 | 1273 | 257.65 |
| HUN | 92249 | 0.0033 | 0.088 | 0.226 | 0.0013 | 3651.51 | 5.97 | 76.0 | 99.7% | 99.2% | 98.4% | 0.852 | 16726 | 7558.89 |
| IDN | 4841680 | 0.0095 | 0.104 | 0.101 | 0.0161 | 255.82 | 1.74 | 71.7 | 94.7% | 97.6% | 90.0% | 0.709 | 4196 | 783.59 |
| IND | 24237733 | 0.0139 | 0.136 | 0.088 | 0.0086 | 119.35 | 0.96 | 69.7 | 81.4% | 90.4% | 58.6% | 0.617 | 2099 | 385.54 |
| IRL | 62473 | 0.0028 | 0.064 | 0.184 | 0.0046 | 27664.18 | 26.69 | 82.3 | 99.7% | 99.6% | 99.2% | 0.852 | 80482 | 35897.73 |
| IRN | 1551502 | 0.0068 | 0.060 | 0.147 | 0.0086 | 1343.47 | 1.17 | 76.7 | 99.0% | 98.4% | 95.5% | 0.572 | 6980 | 1044.09 |
| IRQ | 1103987 | 0.0117 | 0.104 | 0.222 | 0.0161 | 631.62 | 1.85 | 70.6 | 95.6% | 97.1% | 73.3% | 0.577 | 5687 | 2732.12 |
| ISL | 4070 | 0.0019 | 0.087 | 0.219 | 0.0025 | 33164.69 | 19.21 | 82.6 | 98.2% | 99.5% | 99.2% | 0.852 | 69572 | 27027.2 |
| ISR | 169767 | 0.0028 | 0.086 | 0.134 | 0.0046 | 15746.6 | 14.75 | 82.8 | *99.0%* | 99.2% | 98.7% | 0.852 | 43603 | 22172.81 |
| ITA | 461610 | 0.0024 | 0.078 | 0.196 | 0.0025 | 12686.43 | 10.97 | 83.2 | 99.9% | 99.2% | 98.7% | 0.852 | 33220 | 18330.82 |
| JAM | 47487 | 0.0127 | 0.098 | 0.156 | 0.0161 | 863.13 | 2.04 | 74.5 | 99.7% | 99.1% | 90.1% | 0.558 | 5782 | 2402.23 |
| JOR | 214646 | 0.0088 | 0.178 | 0.246 | 0.0086 | 884.51 | 1.90 | 74.5 | 99.7% | 98.0% | 93.4% | 0.829 | 4426 | 5749.62 |
| JPN | 953388 | 0.0015 | 0.057 | 0.134 | 0.0013 | 21038.68 | 13.55 | 84.4 | 99.9% | 99.4% | 99.2% | 0.852 | 40802 | 22789.57 |
| KAZ | 388504 | 0.0054 | 0.052 | 0.175 | 0.0161 | 691.73 | 3.65 | 73.2 | 99.9% | 99.6% | 93.0% | 0.615 | 9750 | 4287.45 |
| KEN | 1469068 | 0.0197 | 0.086 | 0.129 | 0.0161 | 164.84 | 0.94 | 66.7 | 70.2% | 96.7% | 62.0% | 0.555 | 2004 | 784.31 |
| KGZ | 154868 | 0.0068 | 0.104 | 0.126 | 0.0161 | 115.74 | 0.63 | 71.6 | 99.8% | 98.8% | 94.9% | 0.661 | 1323 | 889.01 |
| KHM | 366329 | 0.0124 | 0.104 | 0.093 | 0.0161 | 238.42 | 0.79 | 69.8 | 89.0% | 96.7% | 75.4% | 0.774 | 1713 | 324.17 |
| KIR | 3234 | 0.0143 | 0.100 | 0.113 | 0.0161 | 395.73 | 0.84 | 68.4 | 91.9% | 98.7% | 87.0% | 0.544 | 1695 | 477.87 |
| KOR | 379563 | 0.0017 | 0.067 | 0.090 | 0.0025 | 11179.82 | 10.94 | 83.2 | 100.0% | 99.2% | 97.7% | 0.852 | 31846 | 14297.42 |
| KWT | 58281 | 0.0058 | 0.104 | 0.224 | 0.0086 | 6804.71 | 8.82 | 75.5 | 99.9% | 99.2% | 89.6% | 0.852 | 28339 | *15506.18* |
| LAO | 166996 | 0.0166 | 0.104 | 0.158 | 0.0161 | 129.46 | 1.25 | 67.9 | 64.4% | 72.9% | 55.6% | 0.562 | 2625 | 591.68 |
| LBN | 117336 | 0.0063 | 0.090 | 0.182 | 0.0086 | 2045.37 | 2.04 | 78.9 | 98.2% | 98.0% | 93.4% | 0.573 | 7668 | 3777.76 |
| LBR | 158297 | 0.0242 | 0.120 | 0.169 | 0.0161 | 94.7 | 0.38 | 64.1 | 84.4% | 98.0% | 81.9% | 0.637 | 669 | 105.07 |
| LBY | 126282 | 0.0088 | 0.080 | 0.167 | 0.0161 | 555.91 | 1.67 | 72.9 | 99.9% | 95.8% | 87.1% | 0.573 | 6055 | 4709.2 |
| LCA | 2196 | 0.0112 | 0.098 | 0.094 | 0.0161 | 1455.19 | 3.54 | 76.2 | 100.0% | 99.1% | 91.9% | 0.557 | 11803 | 2289.19 |
| LKA | 339036 | 0.0058 | 0.070 | 0.115 | 0.0161 | 363.68 | 1.67 | 77.0 | 99.5% | 99.1% | 94.4% | 0.529 | 3852 | 1582.69 |
| LSO | 56789 | 0.0279 | 0.120 | 0.320 | 0.0161 | 266.32 | 0.49 | 54.3 | 86.6% | 96.8% | 77.6% | 0.561 | 1117 | 533.96 |
| LTU | 29209 | 0.0028 | 0.054 | 0.153 | 0.0013 | 4996.81 | 7.36 | 76.1 | 100.0% | 99.3% | 98.4% | 0.852 | 19553 | 10278.69 |
| LUX | 6362 | 0.0034 | 0.096 | 0.199 | 0.0013 | 32806.18 | 35.32 | 82.4 | 99.9% | 99.6% | 99.2% | 0.852 | 115839 | 102556.91 |
| LVA | 20890 | 0.0032 | 0.054 | 0.154 | 0.0013 | 4098.79 | 6.55 | 75.2 | 99.9% | 95.0% | 94.1% | 0.852 | 17739 | 9270.83 |
| MAR | 681840 | 0.014 | 0.134 | 0.182 | 0.0161 | 401.05 | 1.42 | 76.7 | 86.6% | 87.1% | 51.4% | 0.619 | 3364 | 1269.36 |
| MDA | 41131 | 0.0069 | 0.087 | 0.172 | 0.0013 | 726.85 | 2.00 | 71.9 | 99.7% | 99.3% | 96.3% | 0.663 | 4458 | 5115.44 |
| MDG | 851999 | 0.0165 | 0.120 | 0.092 | 0.0161 | 29.67 | 0.30 | 67.0 | 46.1% | 84.9% | 50.4% | 0.513 | 540 | 88.4 |
| MDV | 7168 | 0.0058 | 0.062 | 0.155 | 0.0161 | 2792.47 | 2.87 | 78.9 | 99.5% | 99.3% | 97.8% | 0.852 | 15149 | 2077.23 |
| MEX | 2224071 | 0.0068 | 0.070 | 0.124 | 0.0025 | 1591.76 | 3.38 | 75.1 | 96.6% | 98.6% | 94.6% | 0.556 | 10024 | 6871.65 |
| MKD | 22658 | 0.0041 | 0.087 | 0.161 | 0.0013 | 1227.33 | 2.51 | 75.8 | 100.0% | 99.4% | 95.7% | 0.545 | 6044 | 5917.26 |
| MLI | 787496 | 0.0197 | 0.120 | 0.161 | 0.0161 | 56.82 | 0.49 | 59.3 | 67.3% | 82.7% | 42.3% | 0.551 | 907 | 94.87 |
| MLT | 4302 | 0.003 | 0.071 | 0.167 | 0.0013 | 10691.81 | 9.78 | 82.6 | 99.7% | 99.3% | 98.8% | 0.852 | 30833 | 12854.82 |
| MMR | 947677 | 0.0141 | 0.104 | 0.113 | 0.0161 | 111.02 | 0.76 | 67.1 | 60.2% | 92.6% | 69.8% | 0.559 | 1302 | *496.32* |
| MNE | 7418 | 0.0036 | 0.087 | 0.144 | 0.0025 | 2321.65 | 3.14 | 76.9 | 98.8% | 91.5% | 87.4% | 0.548 | 8903 | 3761.8 |
| MNG | 76704 | 0.0052 | 0.104 | 0.116 | 0.0161 | 371.2 | 1.81 | 69.9 | 99.3% | 99.0% | 90.6% | 0.536 | 4245 | 1807.12 |
| MOZ | 1099017 | 0.0217 | 0.120 | 0.142 | 0.0161 | 67.21 | 0.28 | 60.9 | 73.0% | 93.4% | 61.2% | 0.523 | 488 | 187.27 |
| MRT | 147274 | 0.022 | 0.120 | 0.169 | 0.0161 | 106.3 | 0.86 | 64.9 | 69.3% | 92.0% | 59.6% | 0.56 | 1954 | 391.46 |
| MUS | 12965 | 0.0103 | 0.120 | 0.164 | 0.0086 | 2132.37 | 3.48 | 74.2 | 99.8% | 99.5% | 95.9% | 0.573 | 11090 | 4292.35 |
| MWI | 614450 | 0.0163 | 0.105 | 0.187 | 0.0161 | 49.24 | 0.38 | 64.3 | 89.8% | 98.9% | 52.4% | 0.482 | 378 | 238.81 |
| MYS | 527284 | 0.0055 | 0.104 | 0.168 | 0.0161 | 1226.37 | 4.10 | 76.2 | 99.6% | 99.5% | 92.3% | 0.561 | 11213 | 6507.72 |
| NAM | 70009 | 0.0147 | 0.120 | 0.218 | 0.0161 | 1194.36 | 1.86 | 63.7 | 88.2% | 97.7% | 84.9% | 0.548 | 5100 | 3604.63 |
| NER | 1022826 | 0.0196 | 0.120 | 0.127 | 0.0161 | 51.18 | 0.34 | 62.4 | 39.1% | 82.7% | 33.6% | 0.515 | 554 | 156.11 |
| NGA | 7377285 | 0.0222 | 0.114 | 0.123 | 0.0161 | 136.91 | 1.03 | 54.7 | 43.3% | 78.3% | 63.0% | 0.558 | 2230 | 193.98 |
| NIC | 134045 | 0.0108 | 0.098 | 0.149 | 0.0161 | 363.84 | 0.95 | 74.5 | 96.0% | 96.4% | 87.6% | 0.63 | 1920 | 2453.02 |
| NLD | 171751 | 0.0023 | 0.074 | 0.191 | 0.0025 | 27070.87 | 17.19 | 82.0 | *99.0%* | 99.6% | 99.2% | 0.852 | 52491 | 30442.73 |
| NOR | 58756 | 0.0024 | 0.058 | 0.218 | 0.0025 | 45023.2 | 21.55 | 82.9 | 99.2% | 99.6% | 99.3% | 0.852 | 75700 | 62010.64 |
| NPL | 562041 | 0.0175 | 0.053 | 0.141 | 0.0161 | 96.19 | 0.62 | 70.8 | 77.2% | 95.4% | 71.0% | 0.585 | 1201 | 398.52 |
| NZL | 59614 | 0.0027 | 0.075 | 0.203 | 0.0025 | 20142.74 | 13.93 | 81.7 | 96.4% | 98.6% | 94.3% | 0.852 | 42166 | 22627.04 |
| OMN | 91892 | 0.0056 | 0.092 | 0.208 | 0.0086 | 1901.4 | 5.48 | 77.9 | 98.6% | 99.4% | 93.2% | 0.852 | 16529 | 9879.04 |
| PAK | 5994075 | 0.0306 | 0.084 | 0.174 | 0.0161 | 67.29 | 0.64 | 67.3 | 71.0% | 90.2% | 56.4% | 0.622 | 1349 | 127.8 |
| PAN | 79013 | 0.0077 | 0.098 | 0.183 | 0.0074 | 4211.81 | 4.82 | 78.5 | 92.9% | 97.9% | 93.2% | 0.852 | 15831 | 13510.63 |
| PER | 574091 | 0.0071 | 0.088 | 0.150 | 0.0161 | 1002.32 | 2.59 | 76.7 | 94.4% | 96.4% | 92.3% | 0.707 | 6958 | 5161.89 |
| PHL | 2177879 | 0.0104 | 0.133 | 0.098 | 0.0086 | 313.34 | 1.52 | 71.2 | 84.4% | 97.3% | 86.5% | 0.629 | 3512 | 956.66 |
| PNG | 232188 | 0.0161 | 0.100 | 0.135 | 0.0161 | 122.35 | 1.30 | 64.5 | 56.4% | 73.2% | 62.2% | 0.552 | 2887 | 590.05 |
| POL | 374676 | 0.0023 | 0.073 | 0.207 | 0.0013 | 3447.89 | 5.89 | 77.9 | 99.8% | 98.9% | 95.6% | 0.852 | 15689 | 7719.01 |
| PRK | 355180 | 0.0085 | 0.104 | 0.085 | 0.0161 | 60.62 | 0.41 | 72.3 | 99.5% | 99.0% | 93.9% | 0.526 | 1300 | *201.16* |
| PRT | 80574 | 0.0025 | 0.077 | 0.158 | 0.0013 | 9089.25 | 7.98 | 80.7 | 99.9% | 99.4% | 98.7% | 0.852 | 23287 | 11354.27 |
| PRY | 143157 | 0.0105 | 0.081 | 0.162 | 0.0161 | 1063.05 | 2.17 | 74.3 | 97.7% | 97.9% | 91.9% | 0.613 | 5333 | 5763.73 |
| QAT | 26312 | 0.0054 | 0.045 | 0.227 | 0.0161 | 7037.7 | 16.52 | 80.2 | 100.0% | 97.6% | 92.1% | 0.852 | 62919 | 79296.78 |
| ROU | 191577 | 0.0032 | 0.087 | 0.164 | 0.0013 | 2334.84 | 4.95 | 75.5 | 94.8% | 89.0% | 74.6% | 0.553 | 12867 | 10091.19 |
| RUS | 1857519 | 0.0038 | 0.086 | 0.128 | 0.0013 | 2008.99 | 4.00 | 73.1 | 99.7% | 99.3% | 95.4% | 0.548 | 11512 | 6810.22 |
| RWA | 390282 | 0.0169 | 0.120 | 0.121 | 0.0161 | 92.2 | 0.46 | 69.0 | 94.3% | 99.4% | 44.5% | 0.542 | 816 | 281.25 |
| SAU | 599706 | 0.005 | 0.040 | 0.210 | 0.0086 | 4756 | 7.32 | 75.1 | 99.4% | 98.8% | 93.5% | 0.852 | 23266 | 15404.51 |
| SDN | 1338967 | 0.0226 | 0.134 | 0.253 | 0.0161 | 82.69 | 0.30 | 65.3 | 77.7% | 87.1% | 60.3% | 0.564 | 777 | 69.31 |
| SEN | 544018 | 0.0197 | 0.120 | 0.142 | 0.0161 | 108.99 | 0.76 | 67.9 | 74.5% | 97.8% | 56.8% | 0.61 | 1430 | 555.62 |
| SGP | 50059 | 0.002 | 0.104 | 0.184 | 0.0013 | 11223.13 | 19.37 | 83.5 | 99.6% | 99.2% | 99.0% | 0.852 | 65641 | 45147.3 |
| SLB | 21098 | 0.0101 | 0.100 | 0.112 | 0.0161 | 234.85 | 1.09 | 73.0 | 86.2% | 95.2% | 80.3% | 0.545 | 2463 | 551.4 |
| SLE | 255448 | 0.0237 | 0.120 | 0.173 | 0.0161 | 81.07 | 0.31 | 54.7 | 86.9% | 96.0% | 86.9% | 0.517 | 527 | 91.69 |
| SLV | 117747 | 0.0101 | 0.096 | 0.109 | 0.0161 | 776.12 | 1.71 | 73.3 | 99.9% | 97.9% | 91.6% | 0.554 | 4187 | *3057.91* |
| SOM | 621537 | 0.0268 | 0.120 | 0.176 | 0.0161 | 32.87 | 0.28 | 57.4 | 31.9% | 49.0% | 12.3% | 0.577 | 338 | 114.21 |
| SRB | 84123 | 0.0044 | 0.120 | 0.143 | 0.0013 | 1962.37 | 3.16 | 75.7 | 99.9% | 99.5% | 94.9% | 0.545 | 7392 | 3682.68 |
| SSD | 385503 | 0.0288 | 0.120 | 0.250 | 0.0161 | 34.68 | 0.22 | 57.8 | 19.4% | 58.7% | 24.1% | 0.564 | 310 | 94.4 |
| STP | 6645 | 0.0126 | 0.120 | 0.196 | 0.0161 | 226.14 | 1.06 | 70.4 | 96.8% | 99.3% | 86.4% | 0.59 | 1980 | 608.51 |
| SUR | 10694 | 0.0112 | 0.098 | 0.101 | 0.0161 | 1880.94 | 2.14 | 71.7 | 98.4% | 96.6% | 72.2% | 0.563 | 6191 | 2364.89 |
| SVK | 57084 | 0.0028 | 0.091 | 0.158 | 0.0013 | 4871.41 | 7.05 | 77.5 | 98.0% | 99.2% | 98.4% | 0.852 | 19281 | 9806.65 |
| SVN | 20171 | 0.0025 | 0.073 | 0.175 | 0.0013 | 9077.38 | 9.04 | 81.3 | 99.8% | 99.3% | 99.2% | 0.852 | 26037 | 13294.53 |
| SWE | 118087 | 0.0024 | 0.058 | 0.192 | 0.0025 | 29220.73 | 17.16 | 83.0 | *99.0%* | 99.1% | 98.7% | 0.852 | 51443 | 32619.23 |
| SWZ | 30241 | 0.0132 | 0.120 | 0.383 | 0.0161 | 664.36 | 1.57 | 60.2 | 88.3% | 99.1% | 81.0% | 0.516 | 4010 | 2011.76 |
| SYC | 1626 | 0.0095 | 0.120 | 0.174 | 0.0161 | 2733.91 | 4.22 | 73.9 | 99.0% | 99.2% | 93.8% | 0.852 | 16389 | 5382.21 |
| SYR | 426043 | 0.0109 | 0.104 | 0.174 | 0.0161 | 384.05 | 1.32 | 72.7 | 96.2% | 90.4% | 70.3% | 0.527 | 2807 | *1092.11* |
| TCD | 647881 | 0.0275 | 0.120 | 0.169 | 0.0161 | 48.18 | 0.39 | 54.2 | 24.3% | 71.9% | 38.4% | 0.546 | 686 | 117.02 |
| TGO | 259879 | 0.0224 | 0.120 | 0.092 | 0.0161 | 91.69 | 0.51 | 61.0 | 69.4% | 94.8% | 62.0% | 0.545 | 893 | 217.3 |
| THA | 724587 | 0.0058 | 0.127 | 0.143 | 0.0086 | 763.92 | 2.97 | 77.2 | 99.1% | 98.8% | 94.2% | 0.558 | 7815 | 6791.59 |
| TJK | 280780 | 0.009 | 0.104 | 0.085 | 0.0161 | 115.14 | 0.49 | 71.1 | 94.8% | 95.1% | 68.7% | 0.635 | 873 | 362.99 |
| TKM | 139062 | 0.0086 | 0.104 | 0.199 | 0.0161 | 1447.5 | 3.14 | 68.2 | 100.0% | 99.3% | 96.4% | 0.545 | 7724 | 2321.26 |
| TLS | 37396 | 0.013 | 0.104 | 0.109 | 0.0161 | 187.11 | 0.75 | 69.5 | 56.7% | 87.2% | 61.7% | 0.59 | 1561 | 302.97 |
| TON | 2525 | 0.0077 | 0.100 | 0.131 | 0.0161 | 598.62 | 2.03 | 70.9 | 98.3% | 98.9% | 94.7% | 0.562 | 5151 | 1392.07 |
| TTO | 18148 | 0.0091 | 0.098 | 0.258 | 0.0086 | 4103.68 | 5.79 | 73.5 | 100.0% | 99.9% | 97.4% | 0.852 | 16637 | 7573.99 |
| TUN | 203694 | 0.0108 | 0.134 | 0.167 | 0.0086 | 570.8 | 1.60 | 76.7 | 99.5% | 96.1% | 81.3% | 0.52 | 3324 | 3354.31 |
| TUR | 1317556 | 0.0044 | 0.124 | 0.115 | 0.0161 | 1090.11 | 3.45 | 77.7 | 97.0% | 97.5% | 90.5% | 0.8 | 9151 | 9283.37 |
| TZA | 2052007 | 0.0188 | 0.166 | 0.170 | 0.0086 | 69.01 | 0.59 | 65.5 | 63.5% | 98.5% | 54.5% | 0.514 | 1080 | 325.63 |
| UGA | 1613703 | 0.0178 | 0.066 | 0.156 | 0.0161 | 53.15 | 0.47 | 63.4 | 74.2% | 98.7% | 61.9% | 0.488 | 949 | 156.19 |
| UKR | 425822 | 0.0045 | 0.087 | 0.168 | 0.0086 | 615.96 | 1.68 | 71.8 | 99.9% | 99.3% | 93.8% | 0.615 | 3688 | 2421.21 |
| URY | 47931 | 0.0047 | 0.090 | 0.145 | 0.0013 | 6339.93 | 5.80 | 77.9 | 100.0% | 98.0% | 94.8% | 0.852 | 17681 | 7271.49 |
| USA | 3900750 | 0.003 | 0.096 | 0.252 | 0.0013 | 66504.44 | 20.47 | 78.8 | 99.1% | 98.9% | 98.8% | 0.852 | 65254 | 35993.89 |
| UZB | 702638 | 0.0065 | 0.104 | 0.146 | 0.0161 | 201.55 | 0.88 | 71.7 | 100.0% | 99.6% | 94.2% | 0.63 | 1736 | 1015.41 |
| VCT | 1578 | 0.0121 | 0.098 | 0.150 | 0.0161 | 952.01 | 3.00 | 72.5 | 98.6% | 99.1% | 98.1% | 0.555 | 7474 | 2496.36 |
| VEN | 527519 | 0.0094 | 0.097 | 0.232 | 0.0161 | 654.21 | 0.86 | 72.1 | 99.1% | 98.7% | 75.0% | 0.559 | 2299 | 646.68 |
| VNM | 1609918 | 0.0078 | 0.065 | 0.095 | 0.0161 | 345.04 | 1.31 | 75.4 | 93.8% | 96.8% | 76.8% | 0.599 | 3415 | 2096.25 |
| VUT | 8615 | 0.0111 | 0.100 | 0.112 | 0.0161 | 222.19 | 1.35 | 70.5 | 89.4% | 91.0% | 71.2% | 0.553 | 3156 | 970.09 |
| WSM | 4808 | 0.0088 | 0.100 | 0.121 | 0.0161 | 563.68 | 1.82 | 73.3 | 88.9% | 95.9% | 64.1% | 0.562 | 4238 | 1077.98 |
| YEM | 864766 | 0.0237 | 0.104 | 0.165 | 0.0161 | 57.63 | 0.35 | 66.1 | 44.7% | 67.6% | 30.6% | 0.577 | 713 | 98.64 |
| ZAF | 1184994 | 0.0164 | 0.124 | 0.285 | 0.0086 | 1568.79 | 2.41 | 64.1 | 96.7% | 96.1% | 82.1% | 0.602 | 5978 | 2983.47 |
| ZMB | 621584 | 0.0148 | 0.120 | 0.210 | 0.0161 | 149.83 | 0.54 | 63.9 | 80.4% | 97.3% | 67.1% | 0.465 | 1272 | 379.28 |
| ZWE | 442285 | 0.016 | 0.120 | 0.273 | 0.0161 | 314 | 0.65 | 61.5 | 86.0% | 94.3% | 74.1% | 0.532 | 1293 | 321.85 |

### A2.2 Scenario Analysis

**Table D: Changes to base case parameter assumptions used in scenario analysis.**

| **Scenario** | **Change to input parameter compared to base case analysis** |
| --- | --- |
| 80% Vaccine Efficacy against GBS-preterm | *VE_GBS-preterm_* = 80%; proportion of preterm births protected among effectively vaccinated mothers based on country-specific estimates |
| Two-dose regimen | *N*_doses_ = 2 |
| 60% Vaccine Efficacy | *VE_iGBS_* = 60%; *VE_GBS-stillbirth_* = 60% |
| 90% Vaccine Efficacy | *VE_iGBS_* = 90%; *VE_GBS-stillbirth_* = 90% |
| High vaccine coverage | *V_coverage_* based on country-specific ANC1 coverage estimates |
| High CFR with no Skilled Birth Attendance | Adjusted CFR for EOGBS assumes 90% case fatality among EOGBS cases without Skilled Birth Attendance (SBA) as a proxy for access to healthcare: *CFR’_EOGBS_ =* *(SBA_coverage_ x* *CFR_EOGBS_) + 0.9 x (1 - SBA_coverage_ )* |
| With mild NDI | Includes excess risk of mild NDI due to iGBS calculated as:  *Risk_mild-NDI-men_ = Risk_any-NDI-men_ - Risk_mod_sev-NDI-men_* where *Risk_any-NDI-men_* is based on the posterior sample from the burden model;  *Risk_mild-NDI-sep_ = Risk_any-NDI-sep_ - Risk_mod_sev-NDI-sep_* where *Risk_any-NDI-sep_* is based on the posterior sample from the burden model |
| No long-term costs | *Cost_disability_ = 0* |
| 50% higher vac delivery costs | 50% increase in country-specific estimates of vaccine delivery costs: *Cost’_disability_ = 1.5 x Cost_disability_* |

### A2.3 Calculation of GBS-stillbirth risk

The risk of stillbirth in colonised and non-colonised branches of the model was calculated by combining the regional estimate of the proportion of stillbirths due to GBS (Table B) with the overall country-specific risk of stillbirth (due to any cause) and the country-specific risk of maternal colonisation (Table C), as follows:

*Risk_stillbirth-no-col_* = *Risk_stillbirth-overall_* * (1 - *Prop_stillbirth-GBS_*)

*Risk_stillbirth-col_* = *Risk_stillbirth-no-col_* + *Risk_stillbirth-overall_* * ((1 - *Prop_stillbirth-GBS_*) / *Risk_maternal-colonisation_*)

Where *Risk_stillbirth-no-col_* and *Risk_stillbirth-col_* are, repectively, the risk of stillbirth among non-colonised and colonised pregnancies; *Prop_stillbirth-GBS_* is the proportion of stillbirths due to GBS (Table B); *Risk_stillbirth-overall_* is the overall country-specific risk of stillbirth due to any cause (Table C); and *Risk_maternal-colonisation_* is the country-specific risk of maternal colonisation (Table C).

### A2.4 Calculation of GBS-associated preterm risk

The risk of preterm birth in the colonised and non-colonised branches of the model was calculated by combining the global odds ratio for prematurity in births to colonised vs non-colonised mothers (Table C) together with the country-specific preterm risk (due to any cause) and country-specific risk of maternal colonisation (Table C), and considering the following two-by-two table:

|  | | Preterm status | |
| --- | --- | --- | --- |
|  |  | Preterm | Term |
| GBS status | Colonised | *A* | *B* |
|  | Not colonised | *C* | *D* |

To find *Risk_preterm-col_* (given by *A*/(*A* + B) ) and *Risk_preterm-no-col_* (given by *C*/(*C* + *D*) ) the following set of equations are solved for *A*, *B*, *C* and *D*:

*A* + *B* + *C* + *D* = 1

*OR_preterm-col_* = (*A*/*B*)/(*C*/*D*)

*Risk*_preterm-overall_ = (*A* + *C*) / (*A* + *B* + *C* + *D*)

*Risk_maternal-colonisation_* = (*A* + *B*) / (*A* + *B* + *C* + *D*)

### A2.5 Proportion of GBS-associated preterm births protected by vaccination

In the scenario where a GBS vaccine also provides protection against GBS-associated preterm births we assume that only a proportion of these preterm births are potentially protected by vaccination depending on the timing of antenatal visits by gestational age. To estimate this proportion, we combined global estimates of the proportion of preterm births occurring by gestational age from Blencowe et al. [22] (Table E) with country-specific estimates for 131 low- and middle-income countries (LMICs) by Baral and colleagues [23] of the proportion of pregnant women who could receive vaccination each week between 24 and 36 weeks of gestational age (Table F). For vaccination at each gestational age, we assumed there would be a two-week delay to establish immunity before protection against preterm birth could occur.

The calculated proportion of preterm births protected amongst effectively vaccinated mothers are reported in Table C. For high-income countries, for which estimates from Baral and colleagues were not available, we assumed a value of 0.852 based on the highest estimated proportion among LMIC countries.

**Table E: Distribution of preterm births by gestational age.** The proportion of preterm births occurring by week of gestational age is based on the global values reported by Blencowe et al. [22]

| **Gestational age (weeks)** | **Proportion of preterm births occurring at this gestational age^a^** | **Proportion of preterm births after this gestational age** |
| --- | --- | --- |
| 24 | 0.013 | 0.987 |
| 25 | 0.013 | 0.974 |
| 26 | 0.013 | 0.961 |
| 27 | 0.013 | 0.948 |
| 28 | 0.026 | 0.922 |
| 29 | 0.026 | 0.896 |
| 30 | 0.026 | 0.870 |
| 31 | 0.026 | 0.844 |
| 32 | 0.063 | 0.781 |
| 33 | 0.063 | 0.717 |
| 34 | 0.239 | 0.478 |
| 35 | 0.239 | 0.239 |
| 36 | 0.239 | 0.000 |

1. Estimates reported by Blencowe and co-workers were reported for sub-groups covering multiple weeks of gestational age, which we divided evenly across the number of weeks in each gestational age band to give approximate estimates by week.

**Table F: Country-specific estimates of the proportion of pregnant women vaccinated by gestation age in weeks.** These estimates were provided by the authors of reference [23] and were calculated using input data on antenatal coverage that was available at the time of their analysis.

| **Country ISO3 code** | **Proportion of pregnant women vaccinated by gestational age in weeks** | | | | | | | | | | | | |
| --- | --- | --- | --- | --- | --- | --- | --- | --- | --- | --- | --- | --- | --- |
|  | **24** | **25** | **26** | **27** | **28** | **29** | **30** | **31** | **32** | **33** | **34** | **35** | **36** |
| MAR | 0.031 | 0.032 | 0.033 | 0.033 | 0.033 | 0.033 | 0.033 | 0.030 | 0.024 | 0.019 | 0.018 | 0.015 | 0.011 |
| AFG | 0.015 | 0.015 | 0.015 | 0.015 | 0.015 | 0.013 | 0.013 | 0.013 | 0.012 | 0.010 | 0.010 | 0.009 | 0.008 |
| AGO | 0.044 | 0.039 | 0.040 | 0.043 | 0.049 | 0.042 | 0.042 | 0.041 | 0.039 | 0.036 | 0.036 | 0.032 | 0.026 |
| ALB | 0.110 | 0.120 | 0.123 | 0.103 | 0.065 | 0.047 | 0.044 | 0.036 | 0.023 | 0.015 | 0.013 | 0.010 | 0.006 |
| ARM | 0.087 | 0.106 | 0.112 | 0.113 | 0.107 | 0.104 | 0.104 | 0.084 | 0.046 | 0.026 | 0.025 | 0.019 | 0.010 |
| AZE | 0.042 | 0.041 | 0.042 | 0.047 | 0.056 | 0.058 | 0.058 | 0.051 | 0.037 | 0.028 | 0.028 | 0.025 | 0.022 |
| BDI | 0.019 | 0.016 | 0.016 | 0.023 | 0.039 | 0.040 | 0.040 | 0.040 | 0.036 | 0.033 | 0.033 | 0.030 | 0.025 |
| BEN | 0.056 | 0.048 | 0.049 | 0.052 | 0.057 | 0.053 | 0.052 | 0.047 | 0.038 | 0.029 | 0.028 | 0.027 | 0.025 |
| BFA | 0.039 | 0.030 | 0.030 | 0.040 | 0.059 | 0.058 | 0.059 | 0.056 | 0.051 | 0.043 | 0.043 | 0.040 | 0.033 |
| BGD | 0.011 | 0.014 | 0.015 | 0.019 | 0.027 | 0.030 | 0.031 | 0.028 | 0.021 | 0.018 | 0.018 | 0.014 | 0.006 |
| BGR | 0.033 | 0.045 | 0.047 | 0.062 | 0.085 | 0.096 | 0.097 | 0.088 | 0.068 | 0.060 | 0.060 | 0.047 | 0.022 |
| BIH | 0.029 | 0.038 | 0.040 | 0.053 | 0.073 | 0.082 | 0.083 | 0.075 | 0.058 | 0.050 | 0.051 | 0.040 | 0.018 |
| BLR | 0.038 | 0.051 | 0.054 | 0.072 | 0.098 | 0.110 | 0.112 | 0.101 | 0.080 | 0.070 | 0.070 | 0.055 | 0.027 |
| BLZ | 0.038 | 0.050 | 0.053 | 0.070 | 0.095 | 0.107 | 0.107 | 0.097 | 0.074 | 0.063 | 0.063 | 0.049 | 0.022 |
| BOL | 0.080 | 0.080 | 0.087 | 0.081 | 0.061 | 0.044 | 0.036 | 0.029 | 0.024 | 0.019 | 0.017 | 0.016 | 0.013 |
| BRA | 0.096 | 0.106 | 0.110 | 0.100 | 0.081 | 0.067 | 0.064 | 0.056 | 0.045 | 0.036 | 0.034 | 0.029 | 0.020 |
| BTN | 0.032 | 0.043 | 0.045 | 0.060 | 0.083 | 0.094 | 0.095 | 0.087 | 0.070 | 0.063 | 0.063 | 0.050 | 0.026 |
| BWA | 0.035 | 0.047 | 0.050 | 0.064 | 0.087 | 0.097 | 0.098 | 0.087 | 0.064 | 0.053 | 0.053 | 0.041 | 0.016 |
| CAF | 0.034 | 0.028 | 0.028 | 0.028 | 0.027 | 0.022 | 0.022 | 0.022 | 0.021 | 0.019 | 0.019 | 0.018 | 0.015 |
| CHN | 0.034 | 0.046 | 0.048 | 0.063 | 0.085 | 0.096 | 0.097 | 0.086 | 0.065 | 0.055 | 0.055 | 0.043 | 0.018 |
| CIV | 0.046 | 0.044 | 0.044 | 0.048 | 0.054 | 0.045 | 0.045 | 0.046 | 0.045 | 0.034 | 0.034 | 0.034 | 0.035 |
| CMR | 0.045 | 0.036 | 0.037 | 0.043 | 0.055 | 0.048 | 0.047 | 0.047 | 0.045 | 0.038 | 0.038 | 0.036 | 0.031 |
| COD | 0.037 | 0.035 | 0.035 | 0.039 | 0.045 | 0.033 | 0.033 | 0.035 | 0.040 | 0.030 | 0.031 | 0.032 | 0.034 |
| COG | 0.046 | 0.035 | 0.036 | 0.049 | 0.075 | 0.077 | 0.078 | 0.073 | 0.064 | 0.057 | 0.057 | 0.050 | 0.038 |
| COL | 0.124 | 0.133 | 0.137 | 0.117 | 0.080 | 0.059 | 0.053 | 0.043 | 0.032 | 0.024 | 0.023 | 0.020 | 0.015 |
| COM | 0.048 | 0.043 | 0.044 | 0.049 | 0.057 | 0.054 | 0.055 | 0.049 | 0.037 | 0.029 | 0.029 | 0.026 | 0.022 |
| CPV | 0.035 | 0.046 | 0.048 | 0.063 | 0.086 | 0.097 | 0.098 | 0.088 | 0.067 | 0.058 | 0.058 | 0.045 | 0.020 |
| CRI | 0.038 | 0.051 | 0.054 | 0.071 | 0.096 | 0.107 | 0.108 | 0.097 | 0.074 | 0.064 | 0.064 | 0.049 | 0.022 |
| CUB | 0.039 | 0.052 | 0.056 | 0.073 | 0.099 | 0.111 | 0.112 | 0.101 | 0.078 | 0.067 | 0.067 | 0.052 | 0.023 |
| DJI | 0.018 | 0.025 | 0.025 | 0.033 | 0.046 | 0.053 | 0.053 | 0.048 | 0.037 | 0.032 | 0.032 | 0.025 | 0.011 |
| DOM | 0.085 | 0.092 | 0.096 | 0.093 | 0.086 | 0.080 | 0.077 | 0.069 | 0.056 | 0.046 | 0.045 | 0.038 | 0.024 |
| DZA | 0.034 | 0.045 | 0.048 | 0.061 | 0.083 | 0.093 | 0.093 | 0.083 | 0.061 | 0.050 | 0.050 | 0.038 | 0.015 |
| ECU | 0.026 | 0.034 | 0.036 | 0.046 | 0.063 | 0.072 | 0.072 | 0.064 | 0.048 | 0.040 | 0.040 | 0.031 | 0.013 |
| EGY | 0.085 | 0.098 | 0.101 | 0.088 | 0.059 | 0.044 | 0.044 | 0.040 | 0.032 | 0.026 | 0.024 | 0.020 | 0.015 |
| ERI | 0.024 | 0.032 | 0.033 | 0.043 | 0.060 | 0.069 | 0.069 | 0.063 | 0.049 | 0.043 | 0.043 | 0.034 | 0.016 |
| ETH | 0.020 | 0.019 | 0.019 | 0.020 | 0.024 | 0.018 | 0.018 | 0.018 | 0.019 | 0.016 | 0.016 | 0.016 | 0.014 |
| FJI | 0.040 | 0.053 | 0.056 | 0.074 | 0.100 | 0.113 | 0.114 | 0.102 | 0.078 | 0.067 | 0.067 | 0.052 | 0.023 |
| FSM | 0.022 | 0.029 | 0.031 | 0.040 | 0.055 | 0.063 | 0.063 | 0.057 | 0.045 | 0.039 | 0.039 | 0.031 | 0.014 |
| GAB | 0.052 | 0.052 | 0.053 | 0.064 | 0.084 | 0.087 | 0.086 | 0.076 | 0.056 | 0.044 | 0.043 | 0.037 | 0.025 |
| GEO | 0.032 | 0.043 | 0.045 | 0.060 | 0.083 | 0.095 | 0.096 | 0.088 | 0.071 | 0.064 | 0.064 | 0.051 | 0.027 |
| GHA | 0.063 | 0.060 | 0.062 | 0.068 | 0.078 | 0.079 | 0.078 | 0.069 | 0.052 | 0.041 | 0.040 | 0.035 | 0.025 |
| GIN | 0.041 | 0.037 | 0.037 | 0.038 | 0.039 | 0.030 | 0.031 | 0.031 | 0.030 | 0.026 | 0.026 | 0.025 | 0.024 |
| GMB | 0.044 | 0.039 | 0.039 | 0.049 | 0.067 | 0.063 | 0.063 | 0.061 | 0.057 | 0.047 | 0.047 | 0.043 | 0.037 |
| GNB | 0.026 | 0.035 | 0.037 | 0.048 | 0.068 | 0.077 | 0.078 | 0.071 | 0.056 | 0.049 | 0.050 | 0.039 | 0.019 |
| GNQ | 0.033 | 0.044 | 0.046 | 0.059 | 0.080 | 0.090 | 0.090 | 0.080 | 0.059 | 0.049 | 0.049 | 0.037 | 0.014 |
| GRD | 0.039 | 0.052 | 0.055 | 0.072 | 0.098 | 0.110 | 0.111 | 0.099 | 0.076 | 0.065 | 0.065 | 0.050 | 0.022 |
| GTM | 0.073 | 0.077 | 0.079 | 0.076 | 0.068 | 0.060 | 0.059 | 0.053 | 0.043 | 0.034 | 0.033 | 0.029 | 0.023 |
| GUY | 0.054 | 0.047 | 0.048 | 0.057 | 0.074 | 0.072 | 0.072 | 0.067 | 0.056 | 0.047 | 0.047 | 0.043 | 0.036 |
| HND | 0.106 | 0.115 | 0.119 | 0.104 | 0.076 | 0.060 | 0.058 | 0.050 | 0.039 | 0.030 | 0.028 | 0.024 | 0.018 |
| HTI | 0.060 | 0.060 | 0.062 | 0.063 | 0.065 | 0.062 | 0.062 | 0.056 | 0.046 | 0.037 | 0.036 | 0.032 | 0.024 |
| IDN | 0.094 | 0.099 | 0.102 | 0.086 | 0.060 | 0.048 | 0.048 | 0.044 | 0.036 | 0.028 | 0.024 | 0.019 | 0.014 |
| IND | 0.034 | 0.030 | 0.031 | 0.036 | 0.045 | 0.048 | 0.048 | 0.041 | 0.028 | 0.020 | 0.020 | 0.018 | 0.014 |
| IRN | 0.041 | 0.054 | 0.057 | 0.075 | 0.100 | 0.112 | 0.112 | 0.099 | 0.074 | 0.061 | 0.061 | 0.047 | 0.019 |
| IRQ | 0.023 | 0.031 | 0.032 | 0.041 | 0.056 | 0.063 | 0.063 | 0.056 | 0.041 | 0.033 | 0.033 | 0.025 | 0.009 |
| JAM | 0.037 | 0.050 | 0.052 | 0.069 | 0.093 | 0.105 | 0.105 | 0.095 | 0.072 | 0.062 | 0.062 | 0.048 | 0.021 |
| JOR | 0.151 | 0.165 | 0.170 | 0.133 | 0.073 | 0.044 | 0.035 | 0.025 | 0.017 | 0.011 | 0.009 | 0.006 | 0.004 |
| KAZ | 0.078 | 0.078 | 0.082 | 0.083 | 0.082 | 0.080 | 0.079 | 0.072 | 0.060 | 0.047 | 0.046 | 0.041 | 0.032 |
| KEN | 0.054 | 0.052 | 0.053 | 0.056 | 0.062 | 0.041 | 0.041 | 0.044 | 0.050 | 0.039 | 0.039 | 0.041 | 0.044 |
| KGZ | 0.080 | 0.092 | 0.097 | 0.097 | 0.090 | 0.085 | 0.085 | 0.073 | 0.052 | 0.039 | 0.038 | 0.031 | 0.020 |
| KHM | 0.089 | 0.090 | 0.101 | 0.096 | 0.072 | 0.048 | 0.032 | 0.022 | 0.019 | 0.015 | 0.014 | 0.011 | 0.007 |
| KIR | 0.026 | 0.035 | 0.037 | 0.049 | 0.067 | 0.076 | 0.077 | 0.070 | 0.055 | 0.048 | 0.048 | 0.038 | 0.018 |
| LAO | 0.010 | 0.013 | 0.013 | 0.017 | 0.023 | 0.027 | 0.027 | 0.024 | 0.018 | 0.015 | 0.015 | 0.012 | 0.005 |
| LBN | 0.037 | 0.050 | 0.052 | 0.068 | 0.091 | 0.102 | 0.102 | 0.091 | 0.067 | 0.055 | 0.055 | 0.042 | 0.016 |
| LBR | 0.079 | 0.082 | 0.085 | 0.081 | 0.072 | 0.063 | 0.062 | 0.057 | 0.049 | 0.041 | 0.040 | 0.034 | 0.024 |
| LBY | 0.035 | 0.047 | 0.050 | 0.064 | 0.087 | 0.097 | 0.097 | 0.086 | 0.064 | 0.053 | 0.053 | 0.040 | 0.016 |
| LCA | 0.037 | 0.050 | 0.053 | 0.069 | 0.094 | 0.105 | 0.106 | 0.095 | 0.073 | 0.062 | 0.062 | 0.048 | 0.021 |
| LKA | 0.031 | 0.042 | 0.044 | 0.059 | 0.082 | 0.093 | 0.094 | 0.087 | 0.070 | 0.063 | 0.063 | 0.051 | 0.026 |
| LSO | 0.057 | 0.055 | 0.056 | 0.060 | 0.066 | 0.058 | 0.058 | 0.057 | 0.056 | 0.046 | 0.046 | 0.043 | 0.037 |
| MDA | 0.088 | 0.100 | 0.104 | 0.102 | 0.091 | 0.082 | 0.079 | 0.069 | 0.053 | 0.041 | 0.040 | 0.034 | 0.023 |
| MDG | 0.029 | 0.024 | 0.024 | 0.028 | 0.036 | 0.026 | 0.027 | 0.028 | 0.030 | 0.028 | 0.028 | 0.027 | 0.026 |
| MDV | 0.167 | 0.171 | 0.176 | 0.135 | 0.074 | 0.044 | 0.030 | 0.017 | 0.011 | 0.007 | 0.006 | 0.004 | 0.003 |
| MEX | 0.039 | 0.052 | 0.055 | 0.072 | 0.098 | 0.110 | 0.111 | 0.099 | 0.076 | 0.065 | 0.065 | 0.051 | 0.023 |
| MKD | 0.037 | 0.049 | 0.052 | 0.069 | 0.094 | 0.106 | 0.107 | 0.097 | 0.076 | 0.066 | 0.067 | 0.052 | 0.025 |
| MLI | 0.025 | 0.024 | 0.024 | 0.027 | 0.031 | 0.027 | 0.028 | 0.027 | 0.026 | 0.022 | 0.022 | 0.020 | 0.017 |
| MMR | 0.041 | 0.034 | 0.034 | 0.038 | 0.044 | 0.043 | 0.043 | 0.041 | 0.037 | 0.030 | 0.030 | 0.029 | 0.027 |
| MNE | 0.032 | 0.042 | 0.045 | 0.059 | 0.081 | 0.091 | 0.092 | 0.083 | 0.065 | 0.056 | 0.056 | 0.044 | 0.021 |
| MNG | 0.034 | 0.046 | 0.048 | 0.064 | 0.088 | 0.100 | 0.101 | 0.093 | 0.074 | 0.065 | 0.066 | 0.052 | 0.026 |
| MOZ | 0.040 | 0.033 | 0.033 | 0.036 | 0.041 | 0.023 | 0.023 | 0.026 | 0.033 | 0.030 | 0.030 | 0.032 | 0.036 |
| MRT | 0.027 | 0.036 | 0.038 | 0.049 | 0.068 | 0.076 | 0.077 | 0.069 | 0.052 | 0.044 | 0.044 | 0.034 | 0.015 |
| MUS | 0.035 | 0.047 | 0.049 | 0.064 | 0.086 | 0.097 | 0.097 | 0.086 | 0.064 | 0.053 | 0.053 | 0.040 | 0.016 |
| MWI | 0.042 | 0.033 | 0.034 | 0.040 | 0.054 | 0.040 | 0.040 | 0.044 | 0.052 | 0.050 | 0.050 | 0.047 | 0.042 |
| MYS | 0.035 | 0.047 | 0.049 | 0.064 | 0.088 | 0.099 | 0.099 | 0.089 | 0.067 | 0.057 | 0.057 | 0.044 | 0.019 |
| NAM | 0.059 | 0.052 | 0.054 | 0.060 | 0.071 | 0.061 | 0.061 | 0.060 | 0.058 | 0.051 | 0.051 | 0.047 | 0.040 |
| NER | 0.028 | 0.021 | 0.021 | 0.026 | 0.035 | 0.030 | 0.030 | 0.031 | 0.032 | 0.026 | 0.026 | 0.026 | 0.025 |
| NGA | 0.034 | 0.027 | 0.027 | 0.029 | 0.033 | 0.025 | 0.025 | 0.027 | 0.029 | 0.022 | 0.022 | 0.022 | 0.022 |
| NIC | 0.077 | 0.080 | 0.083 | 0.080 | 0.073 | 0.065 | 0.064 | 0.058 | 0.048 | 0.041 | 0.040 | 0.036 | 0.030 |
| NPL | 0.039 | 0.036 | 0.037 | 0.045 | 0.061 | 0.065 | 0.065 | 0.058 | 0.042 | 0.033 | 0.033 | 0.028 | 0.020 |
| PAK | 0.034 | 0.033 | 0.034 | 0.033 | 0.030 | 0.028 | 0.028 | 0.027 | 0.023 | 0.017 | 0.017 | 0.016 | 0.014 |
| PER | 0.110 | 0.117 | 0.121 | 0.107 | 0.082 | 0.069 | 0.065 | 0.055 | 0.042 | 0.031 | 0.030 | 0.026 | 0.020 |
| PHL | 0.068 | 0.065 | 0.067 | 0.072 | 0.081 | 0.083 | 0.083 | 0.071 | 0.050 | 0.037 | 0.036 | 0.032 | 0.025 |
| PNG | 0.020 | 0.027 | 0.028 | 0.037 | 0.051 | 0.058 | 0.058 | 0.052 | 0.040 | 0.035 | 0.035 | 0.027 | 0.012 |
| PRK | 0.033 | 0.045 | 0.047 | 0.063 | 0.088 | 0.100 | 0.102 | 0.093 | 0.076 | 0.069 | 0.069 | 0.056 | 0.029 |
| PRY | 0.062 | 0.071 | 0.074 | 0.074 | 0.074 | 0.064 | 0.064 | 0.059 | 0.051 | 0.042 | 0.040 | 0.035 | 0.029 |
| PSE | 0.031 | 0.042 | 0.044 | 0.059 | 0.083 | 0.095 | 0.097 | 0.090 | 0.074 | 0.068 | 0.069 | 0.056 | 0.031 |
| ROU | 0.022 | 0.030 | 0.031 | 0.041 | 0.056 | 0.063 | 0.064 | 0.058 | 0.044 | 0.038 | 0.038 | 0.030 | 0.013 |
| RUS | 0.033 | 0.045 | 0.047 | 0.062 | 0.085 | 0.096 | 0.097 | 0.088 | 0.068 | 0.060 | 0.060 | 0.047 | 0.022 |
| RWA | 0.033 | 0.028 | 0.029 | 0.045 | 0.076 | 0.084 | 0.084 | 0.075 | 0.057 | 0.044 | 0.044 | 0.039 | 0.030 |
| SDN | 0.022 | 0.029 | 0.030 | 0.039 | 0.054 | 0.061 | 0.061 | 0.055 | 0.041 | 0.035 | 0.035 | 0.027 | 0.011 |
| SEN | 0.047 | 0.044 | 0.045 | 0.056 | 0.076 | 0.079 | 0.080 | 0.069 | 0.046 | 0.032 | 0.032 | 0.029 | 0.023 |
| SLB | 0.026 | 0.035 | 0.037 | 0.048 | 0.067 | 0.076 | 0.076 | 0.069 | 0.054 | 0.047 | 0.047 | 0.037 | 0.018 |
| SLE | 0.055 | 0.045 | 0.046 | 0.055 | 0.072 | 0.067 | 0.067 | 0.066 | 0.064 | 0.060 | 0.061 | 0.054 | 0.042 |
| SLV | 0.036 | 0.048 | 0.051 | 0.067 | 0.091 | 0.102 | 0.103 | 0.093 | 0.072 | 0.062 | 0.062 | 0.048 | 0.022 |
| SOM | 0.002 | 0.002 | 0.002 | 0.003 | 0.004 | 0.005 | 0.005 | 0.004 | 0.003 | 0.002 | 0.002 | 0.002 | 0.001 |
| SRB | 0.037 | 0.049 | 0.052 | 0.068 | 0.093 | 0.105 | 0.106 | 0.096 | 0.076 | 0.066 | 0.066 | 0.052 | 0.025 |
| SSD | 0.010 | 0.013 | 0.014 | 0.017 | 0.024 | 0.028 | 0.028 | 0.025 | 0.019 | 0.016 | 0.016 | 0.012 | 0.005 |
| STP | 0.068 | 0.067 | 0.069 | 0.074 | 0.081 | 0.071 | 0.070 | 0.066 | 0.057 | 0.048 | 0.047 | 0.044 | 0.037 |
| SUR | 0.031 | 0.041 | 0.043 | 0.056 | 0.076 | 0.085 | 0.086 | 0.077 | 0.058 | 0.049 | 0.049 | 0.038 | 0.016 |
| SWZ | 0.063 | 0.046 | 0.047 | 0.055 | 0.071 | 0.055 | 0.055 | 0.057 | 0.062 | 0.053 | 0.054 | 0.054 | 0.056 |
| SYR | 0.021 | 0.028 | 0.030 | 0.039 | 0.055 | 0.063 | 0.064 | 0.059 | 0.048 | 0.043 | 0.043 | 0.035 | 0.018 |
| TCD | 0.017 | 0.011 | 0.011 | 0.014 | 0.019 | 0.017 | 0.017 | 0.016 | 0.015 | 0.013 | 0.013 | 0.012 | 0.010 |
| TGO | 0.041 | 0.032 | 0.032 | 0.036 | 0.043 | 0.033 | 0.033 | 0.034 | 0.036 | 0.030 | 0.030 | 0.030 | 0.029 |
| THA | 0.038 | 0.051 | 0.054 | 0.071 | 0.096 | 0.108 | 0.109 | 0.098 | 0.075 | 0.064 | 0.064 | 0.049 | 0.022 |
| TJK | 0.042 | 0.041 | 0.043 | 0.046 | 0.051 | 0.052 | 0.052 | 0.045 | 0.031 | 0.022 | 0.022 | 0.019 | 0.014 |
| TKM | 0.038 | 0.050 | 0.053 | 0.071 | 0.097 | 0.109 | 0.110 | 0.100 | 0.078 | 0.069 | 0.069 | 0.054 | 0.026 |
| TLS | 0.042 | 0.041 | 0.042 | 0.042 | 0.042 | 0.039 | 0.039 | 0.037 | 0.032 | 0.030 | 0.029 | 0.025 | 0.018 |
| TON | 0.036 | 0.048 | 0.051 | 0.066 | 0.090 | 0.101 | 0.102 | 0.091 | 0.069 | 0.058 | 0.058 | 0.045 | 0.019 |
| TUN | 0.029 | 0.039 | 0.041 | 0.055 | 0.078 | 0.089 | 0.090 | 0.083 | 0.069 | 0.062 | 0.063 | 0.051 | 0.028 |
| TUR | 0.138 | 0.143 | 0.149 | 0.120 | 0.073 | 0.048 | 0.038 | 0.029 | 0.023 | 0.017 | 0.013 | 0.010 | 0.007 |
| TZA | 0.044 | 0.036 | 0.036 | 0.042 | 0.052 | 0.040 | 0.040 | 0.043 | 0.048 | 0.041 | 0.041 | 0.041 | 0.040 |
| UGA | 0.047 | 0.039 | 0.040 | 0.045 | 0.056 | 0.045 | 0.045 | 0.049 | 0.058 | 0.055 | 0.055 | 0.052 | 0.045 |
| UKR | 0.051 | 0.061 | 0.064 | 0.065 | 0.063 | 0.062 | 0.064 | 0.057 | 0.042 | 0.035 | 0.035 | 0.031 | 0.024 |
| UZB | 0.064 | 0.072 | 0.075 | 0.078 | 0.078 | 0.077 | 0.078 | 0.069 | 0.051 | 0.039 | 0.039 | 0.033 | 0.023 |
| VCT | 0.040 | 0.054 | 0.057 | 0.075 | 0.102 | 0.114 | 0.115 | 0.103 | 0.080 | 0.068 | 0.068 | 0.053 | 0.024 |
| VEN | 0.037 | 0.049 | 0.052 | 0.068 | 0.092 | 0.104 | 0.104 | 0.094 | 0.071 | 0.061 | 0.061 | 0.047 | 0.021 |
| VNM | 0.048 | 0.050 | 0.052 | 0.060 | 0.076 | 0.080 | 0.081 | 0.071 | 0.051 | 0.037 | 0.036 | 0.033 | 0.027 |
| VUT | 0.019 | 0.025 | 0.026 | 0.033 | 0.046 | 0.053 | 0.053 | 0.048 | 0.037 | 0.031 | 0.032 | 0.025 | 0.011 |
| WSM | 0.033 | 0.044 | 0.046 | 0.060 | 0.082 | 0.092 | 0.093 | 0.083 | 0.063 | 0.053 | 0.053 | 0.041 | 0.017 |
| YEM | 0.021 | 0.019 | 0.019 | 0.019 | 0.017 | 0.018 | 0.018 | 0.017 | 0.016 | 0.013 | 0.013 | 0.013 | 0.012 |
| ZAF | 0.069 | 0.067 | 0.069 | 0.073 | 0.078 | 0.063 | 0.062 | 0.057 | 0.050 | 0.043 | 0.042 | 0.040 | 0.035 |
| ZMB | 0.053 | 0.033 | 0.033 | 0.041 | 0.055 | 0.045 | 0.045 | 0.050 | 0.061 | 0.059 | 0.059 | 0.056 | 0.050 |
| ZWE | 0.044 | 0.036 | 0.036 | 0.049 | 0.074 | 0.075 | 0.075 | 0.070 | 0.060 | 0.047 | 0.047 | 0.045 | 0.042 |

### A2.6 Estimation of acute costs

We are only aware of one study by Schroeder et al.[7] that has directly estimated the healthcare costs of the acute invasive GBS episode. Hence, to estimate these costs we combined this estimate together with the healthcare costs for neonatal and infant sepsis or meningitis from a systematics review, and two recent cost estimates for neonatal bacterial sepsis and meningitis in Mozambique and South Africa.[6,8] These data are summarised in Table G. All costs were inflated to the year 2020 using the World Bank GDP deflator for the reported currency and costs not reported in United States Dollars were converted using average exchange rates.[24,25] We excluded older estimates from before 2005 reported in the systematic review, which were all studies from the United States.

**Table G: Cost data used to extrapolate country-specific estimates of the acute healthcare costs of the invasive GBS episode.**

| **Lead author** | **Country ISO3** | **Study year** | **Average cost (2020 USD)** | **Reference** |
| --- | --- | --- | --- | --- |
| Liao | CHN | 2017 | 1618.13 | [6] |
| Qian | CHN | 2008 | 2325.43 |  |
| Atif | DZA | 2008 | 2260.13 |  |
| Inigo | ESP | 2006 | 23797.47 |  |
| Gard | IND | 2005 | 57.68 |  |
| Tanihara | JPN | 2014 | 25862.87 |  |
| Hartman | USA | 2005 | 98418.97 |  |
| Johnson | USA | 2013 | 109497.62 |  |
| Donovan | USA | 2013 | 119136.88 |  |
| Balamuth | USA | 2013 | 136743.08 |  |
| Balada-Llasat | USA | 2018 | 13875.82 |  |
| Davis | USA | 2011 | 35479.86 |  |
| Davis | USA | 2011 | 59373.58 |  |
| Anh | VNM | 2010 | 233.7 |  |
| Aerts | MOZ | 2020 | 109.08 | [8] |
| Aerts | ZAF | 2020 | 304.62 |  |
| Schroeder | GBR | 2009 | 9675.68 | [7] |

To extrapolate these acute cost estimates across countries we log-transformed the cost data and fitted a linear-model using log-transformed country-specific per capita healthcare expenditure as a predictor, with the resulting fit below. This model was then used to parameterise a log-normal distribution for the acute cost parameter in each country. The mean estimates of the country-specific acute costs are reported in Table C.

*ln (costs) = -0.4466 + 1.2380 x ln (per capita healthcare expenditure)*

**Fitted regression model used to extrapolate acute costs across countries.**

### A2.7 Estimation of vaccine delivery costs

To estimate country-specific vaccine delivery costs (in addition to the price of purchasing the vaccine) we used data on average delivery costs per dose for other maternal vaccinations from a recent systematic review. As for the acute cost data, these estimates were inflated and converted to 2020 USD and are reported in Table H.

**Table H: Cost data from reference [5] used to extrapolate used to extrapolate country-specific estimates of vaccine delivery costs per dose.**

| **Lead author** | **Country ISO3** | **Study year** | **Average cost per dose (2020 USD)** |
| --- | --- | --- | --- |
| Berman | IDN | 1991 | 0.66 |
| Pecenka | MWI | 2017 | 0.57 |
| Zhao | CHN | 2016 | 1.29 |
| Sartori | BRA | 2016 | 2.2 |
| Kim | ZAF | 2014 | 6.75 |
| Skedgel | CAN | 2011 | 5.93 |
| Baguelin | GBR | 2010 | 8.55 |
| van Hoek | GBR | 2016 | 10.7 |
| Fernández-Cano | ESP | 2015 | 10.78 |
| Garcia | ESP | 2016 | 13.24 |
| Giorgakoudi | GBR | 2018 | 14.29 |
| Jit | GBR | 2010 | 16.82 |
| Xu | USA | 2016 | 17.67 |
| Terranella | USA | 2013 | 23.22 |
| Atkins | USA | 2016 | 24.93 |
| Kim | USA | 2017 | 26.74 |
| Skedgel | CAN | 2011 | 41.07 |

To extrapolate the vaccine delivery costs estimates across countries we log-transformed the cost data and fitted a linear-model using log-transformed country-specific per capita gross domestic product as a predictor with the resulting fit below. We then used this model for each country to parameterise a log-normal distribution for the vaccine delivery cost per dose. The resulting estimates for the mean delivery costs in each country are reported in Table C.

*ln (costs) = -6.8002 + 0.8859 x ln (GDP per capita)*

**Fitted regression model used to extrapolate vaccine delivery costs across countries.**

### A2.8 Cost-effectiveness thresholds

As described in the methods section of the main paper, our analysis included two different assumptions about the cost-effectiveness thresholds (CETs) at which a new intervention may be considered cost-effective in each country. Under one normative scenario (main paper Table 1) we assumed a commonly used CET of 1 x GDP per capita. In an alternative scenario we used empirical estimates reported by Ochalek et al. and Woods et al., which aim to reflect the health opportunity cost associated with current healthcare expenditure.

The empirical CETs in 2020 USD used in our analysis for each country are reported in Table C. These values were calculated as follows. For countries included in the analysis by Ochalek and co-workers we used an average across the four different CET estimates reported (corresponding to different methodological approaches DALY1 to DALY4 described in the paper). To adjust the CET to 2020 currency values, we applied the average CET as a % of GDP per capita for each country to the country-specific GDP per capita for 2020. For countries not included in the Ochalek et al. analysis, we instead used the midpoint of the estimated CET ranges reported by Woods et al. and uplifted the estimates to 2020 USD values in line with the change in GDP per capita in each country. For countries that that were not included in either the Ochalek et al. or Woods et al. analyses, we imputed the CET using linear regression against GDP per capita.

## A3 Supplementary Results

**Table I: Annual global and regional incremental impact of GBS vaccination compared with no vaccination for 2020 under high-coverage scenario**

| **Description** | **Central & Southern Asia** | **Eastern & South-Eastern Asia** | **Europe & Northern America** | **Latin America & Caribbean** | **Northern Africa & Western Asia** | **Oceania** | **Sub-Saharan Africa** | **Global^** |
| --- | --- | --- | --- | --- | --- | --- | --- | --- |
| Number of women vaccinated  (millions) | 34.8 | 29.4 | 11.9 | 10.2 | 9.37 | 0.601 | 33 | 129 |
| Vaccine programme costs  (discounted; $ millions) | 177  (166, 197) | 530  (508, 558) | 654  (627, 694) | 184  (179, 189) | 143  (140, 147) | 26  (24.5, 28.4) | 155  (151, 161) | 1,870  (1,830, 1,920) |
| Acute healthcare costs  (discounted; $ millions) | -9.79  (-21.2, -4.61) | -62.8  (-146, -28.1) | -155  (-342, -60.9) | -23  (-50.7, -11.4) | -31.3  (-64.8, -15) | -4.16  (-9.55, -1.61) | -18.2  (-40.9, -8.45) | -320  (-562, -167) |
| Long-term healthcare costs  (discounted; $ millions) | -3.55  (-14, -0.7) | -22.5  (-92.8, -4.45) | -33.3  (-117, -7.22) | -8.71  (-33.9, -1.72) | -11.3  (-41.7, -2.38) | -0.93  (-3.24, -0.198) | -6.4  (-24.3, -1.25) | -95  (-286, -21.4) |
| Total incremental costs  (discounted; $ millions) | 163  (142, 184) | 442  (304, 501) | 463  (221, 587) | 151  (104, 169) | 99.2  (43, 124) | 20.9  (13.7, 24.8) | 130  (94.8, 145) | 1,450  (1,070, 1,660) |
| EOGBS cases  (thousands) | -34.2  (-64.9, -17.6) | -35.5  (-72.2, -16.9) | -3.43  (-6.04, -1.61) | -9.97  (-18.9, -5.22) | -21.7  (-43.9, -10.3) | -0.483  (-1, -0.235) | -63.5  (-131, -30.1) | -169  (-332, -83.9) |
| LOGBS cases  (thousands) | -17.1  (-49.5, -4.41) | -17.6  (-53, -4.47) | -2.03  (-4.27, -0.855) | -6.58  (-22.7, -2.16) | -15.6  (-39.6, -6.33) | -0.315  (-1.63, -0.0991) | -54.8  (-153, -21) | -118  (-286, -50.9) |
| Moderate & severe NDI cases  (thousands) | -4.37  (-12.8, -1.4) | -4.48  (-13.7, -1.4) | -0.262  (-0.581, -0.0992) | -1.46  (-4.44, -0.468) | -3.17  (-9.16, -1.08) | -0.0625  (-0.261, -0.0208) | -10  (-29.1, -3.31) | -24.2  (-67.1, -8.51) |
| GBS deaths  (thousands) | -6.34  (-14.4, -2.69) | -6.48  (-15.6, -2.69) | -0.341  (-0.677, -0.151) | -2.18  (-5.3, -0.892) | -5.72  (-12.9, -2.47) | -0.0967  (-0.393, -0.0274) | -20.3  (-48.2, -8.45) | -42.3  (-90.7, -19.4) |
| GBS stillbirths  (thousands) | -10.6  (-36.8, -2.6) | -3.62  (-12.6, -0.917) | -0.597  (-1.5, -0.209) | -1.46  (-9.13, -0.242) | -1.71  (-4.14, -0.746) | -0.0744  (-0.392, -0.0195) | -14.1  (-28.1, -6.24) | -33  (-80.3, -14.4) |
| QALYs from averted GBS disease  (discounted; thousands) | 224  (99.7, 514) | 235  (102, 565) | 12.7  (5.72, 24.9) | 78  (33.9, 190) | 197  (88, 445) | 3.41  (1.18, 12.5) | 646  (279, 1,530) | 1,420  (661, 3,140) |
| QALYs from averted stillbirths  (discounted; thousands) | 288  (71.3, 995) | 101  (25.3, 346) | 16.8  (5.89, 42.6) | 40.8  (6.83, 251) | 47.4  (20.8, 114) | 2.01  (0.532, 10.5) | 369  (164, 742) | 889  (384, 2,180) |
| QALYs from averted GBS disease  (undiscounted; thousands) | 534  (237, 1,220) | 593  (257, 1,420) | 33.1  (14.9, 64.8) | 196  (85.3, 477) | 490  (219, 1,110) | 8.01  (2.81, 28.8) | 1,420  (614, 3,360) | 3,340  (1,560, 7,370) |
| QALYs from averted stillbirths  (undiscounted; thousands) | 684  (169, 2,360) | 252  (63.4, 865) | 43.4  (15.3, 110) | 103  (17.2, 632) | 117  (51.7, 280) | 4.78  (1.32, 24.1) | 809  (361, 1,630) | 2,070  (890, 5,160) |
| All values are reported to 3 significant figures. Values in brackets are 95% uncertainty ranges.  GBS = Group B Streptococcus; EOGBS = Early-Onset GBS; LOGBS = Late-Onset GBS; NDI = Neurodevelopmental Impairment; QALY = Quality Adjust Life Year.  *in scenario analysis where vaccine is assumed to have 80% VE against GBS associated prematurity  ^global median values do not exactly equal the sum of the regional median values | | | | | | | | |

***
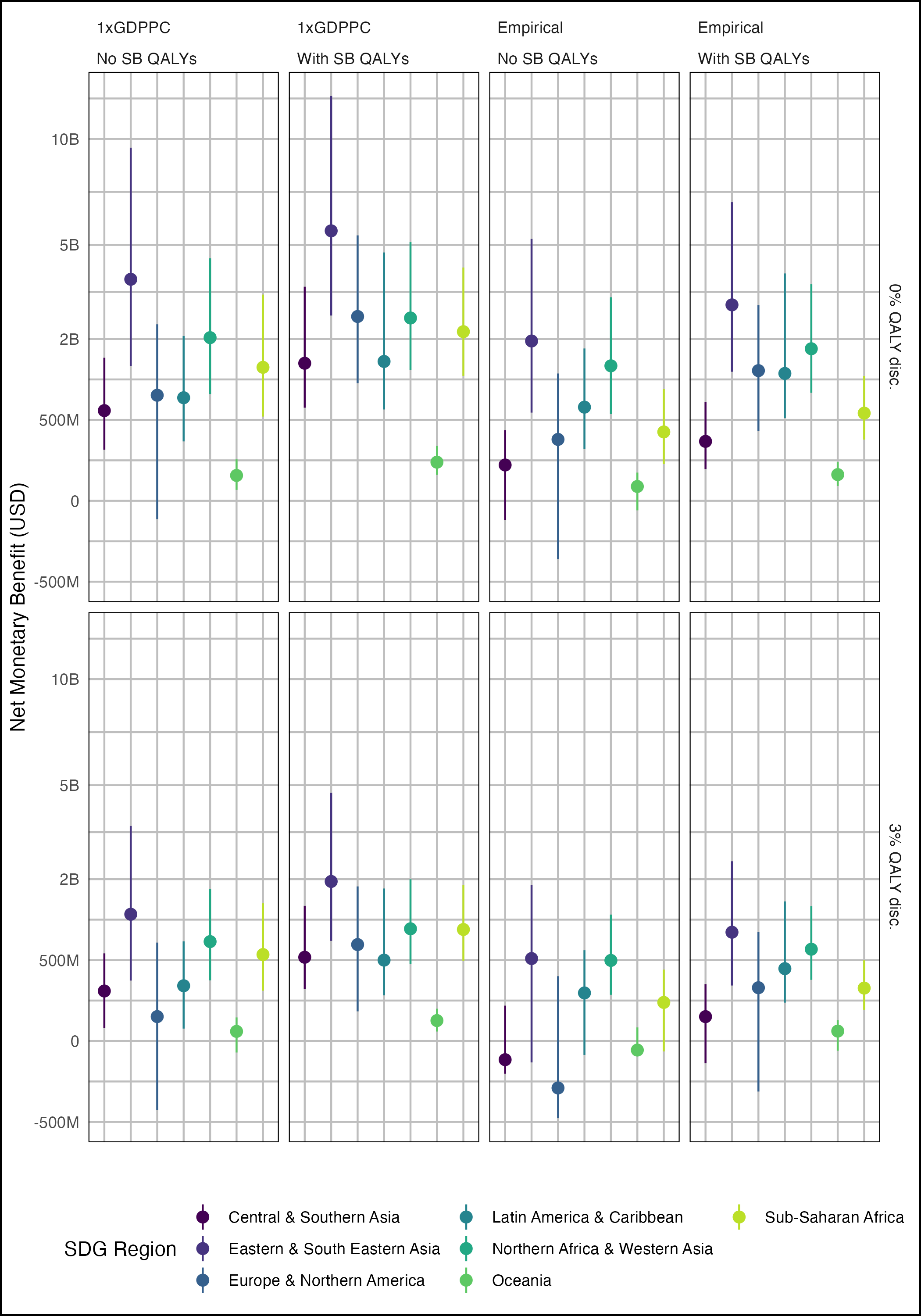
 Fig A:* Regional Net Monetary Benefit of GBS vaccination under different normative assumptions about the discount rate for QALYs, the cost-effectiveness threshold, and whether the value of QALYs for averted stillbirths are included.** M = Millions; B = Billions; CET = Cost-Effectiveness Threshold; GDP = Gross Domestic Product; GDPPC = GDP per capita; QALY = Quality Adjusted Life Year; SB = Stillbirth; SDG = Sustainable Development Goal.

***
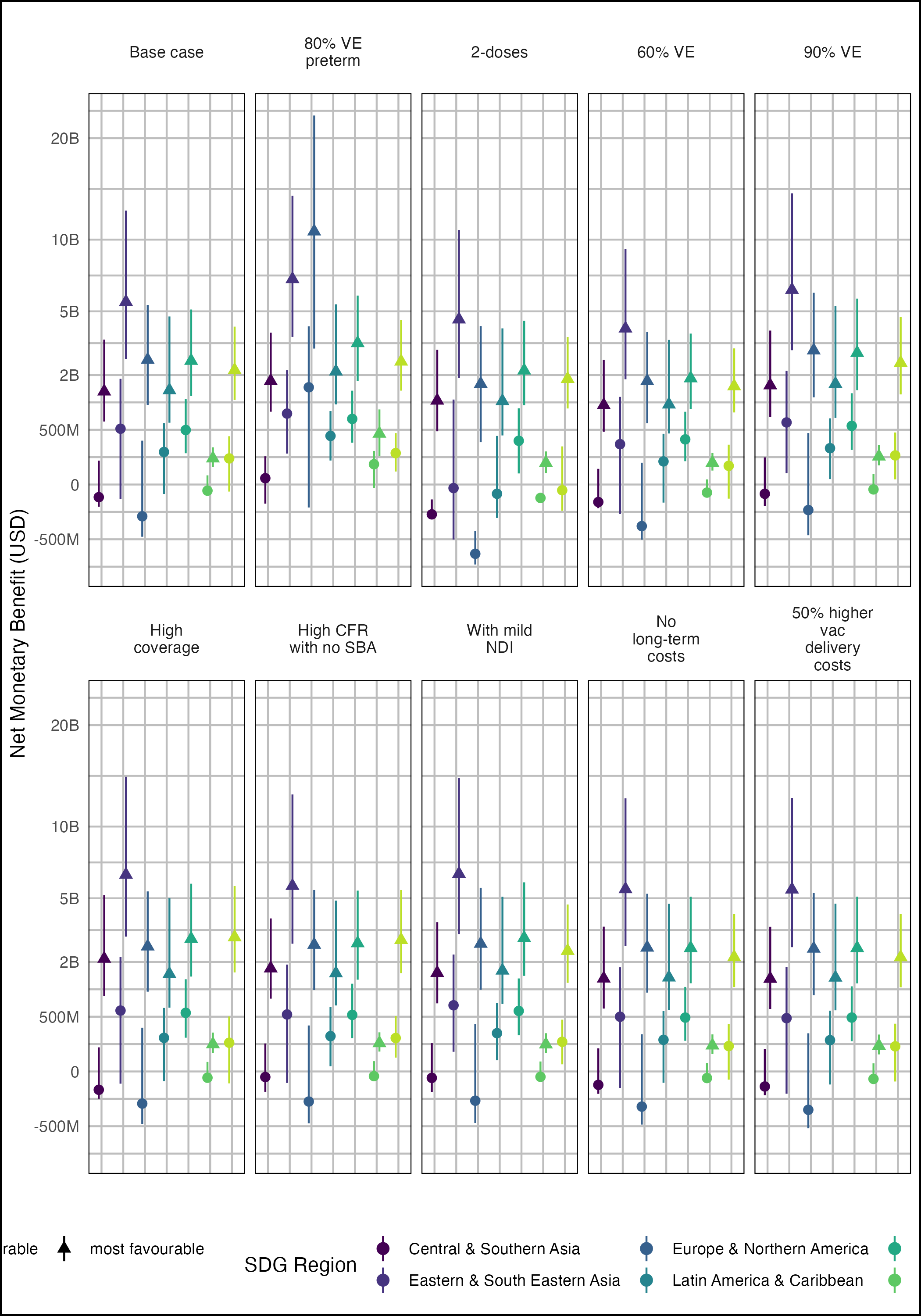
Fig B:* Regional Net Monetary Benefit of different GBS maternal vaccination scenarios under the most and least favourable normative assumptions.** Least-favourable normative assumptions were the use of an empirical CET, 3% discounting of QALYs, and exclusion of stillbirth QALYs. Most-favourable assumptions were the use of 1 x GDP per capita CETs, 0% discounting of QALYs, and inclusion of stillbirth QALYs. M = Millions; B = Billions; CET = Cost-Effectiveness Threshold; GDP = Gross Domestic Product; GDPPC = GDP per capita; QALY = Quality Adjusted Life Year; SB = Stillbirth; SDG = Sustainable Development Goal.

***
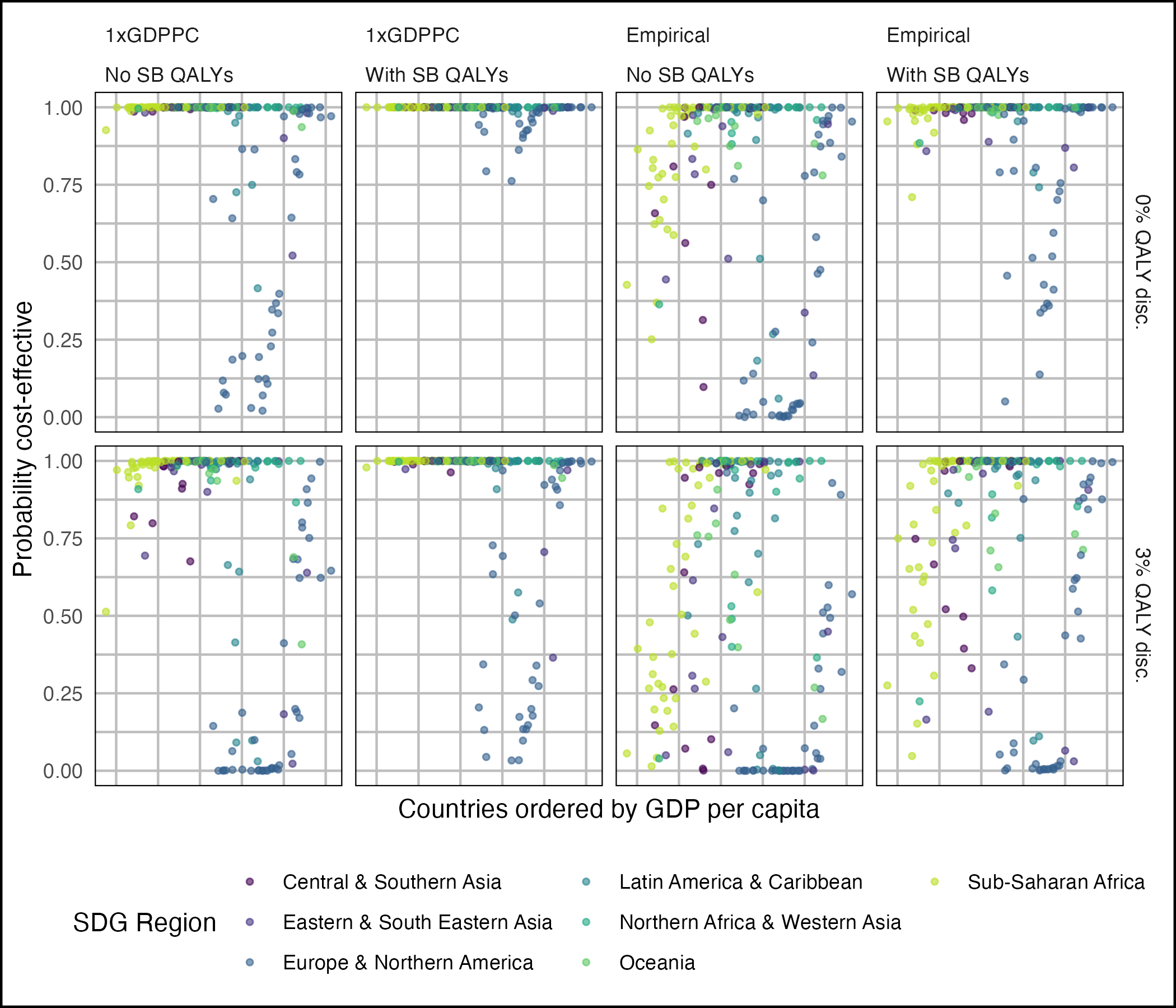
 Fig C:* Probability that GBS maternal vaccination is cost-effective in each country for base case vaccination scenario and different normative assumptions about the discount rate for QALYs, the cost-effectiveness threshold, and whether the value of QALYs for averted stillbirths are included.** M = Millions; B = Billions; CET = Cost-Effectiveness Threshold; GDP = Gross Domestic Product; GDPPC = GDP per capita; QALY = Quality Adjusted Life Year; SB = Stillbirth; SDG = Sustainable Development Goal.

***
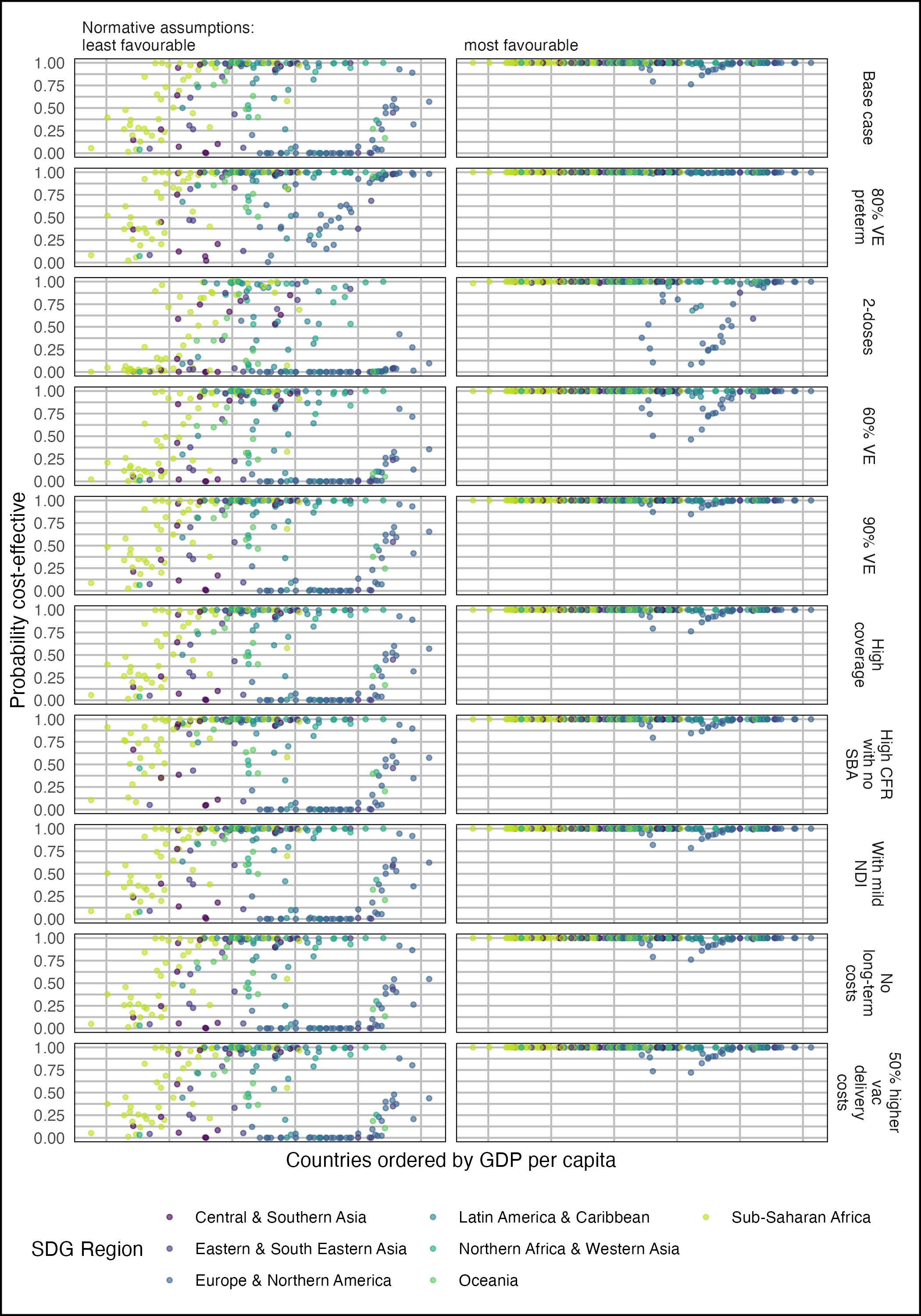
 Fig D:* Probability that GBS maternal vaccination is cost-effective in each country for different vaccination scenarios under most favourable and least favourable normative assumptions.** Least-favourable normative assumptions were the use of an empirical CET, 3% discounting of QALYs, and exclusion of stillbirth QALYs. Most-favourable assumptions were the use of 1 x GDP per capita CETs, 0% discounting of QALYs, and inclusion of stillbirth QALYs. CET = Cost-Effectiveness Threshold; GDP = Gross Domestic Product; GDPPC = GDP per capita; QALY = Quality Adjusted Life Year; SB = Stillbirth; SDG = Sustainable Development Goal.

***
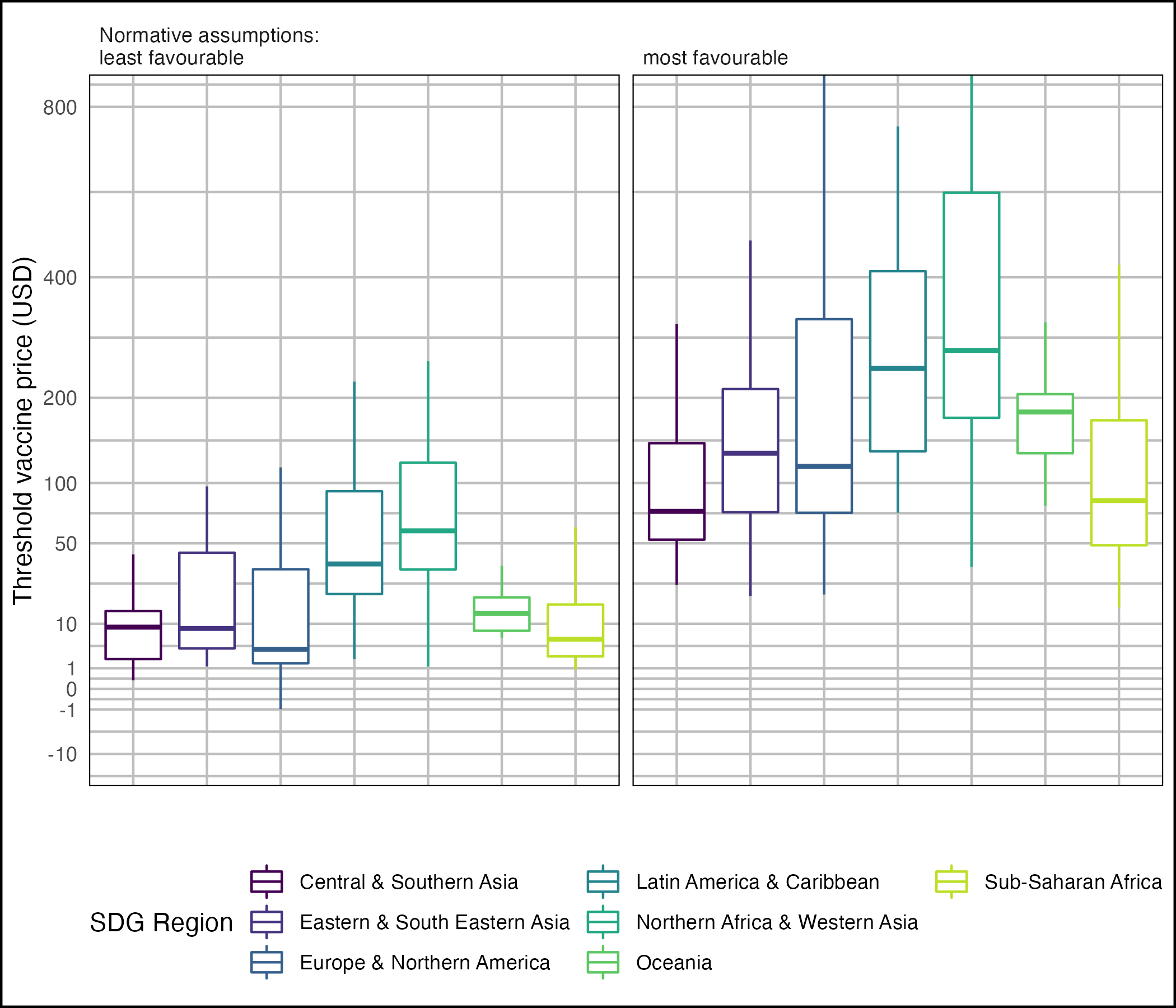
 Fig E:* Distribution of GBS vaccine threshold prices amongst countries within each SDG region under most and least favourable normative assumptions** Threshold vaccine prices above $800 per dose are not shown.

***
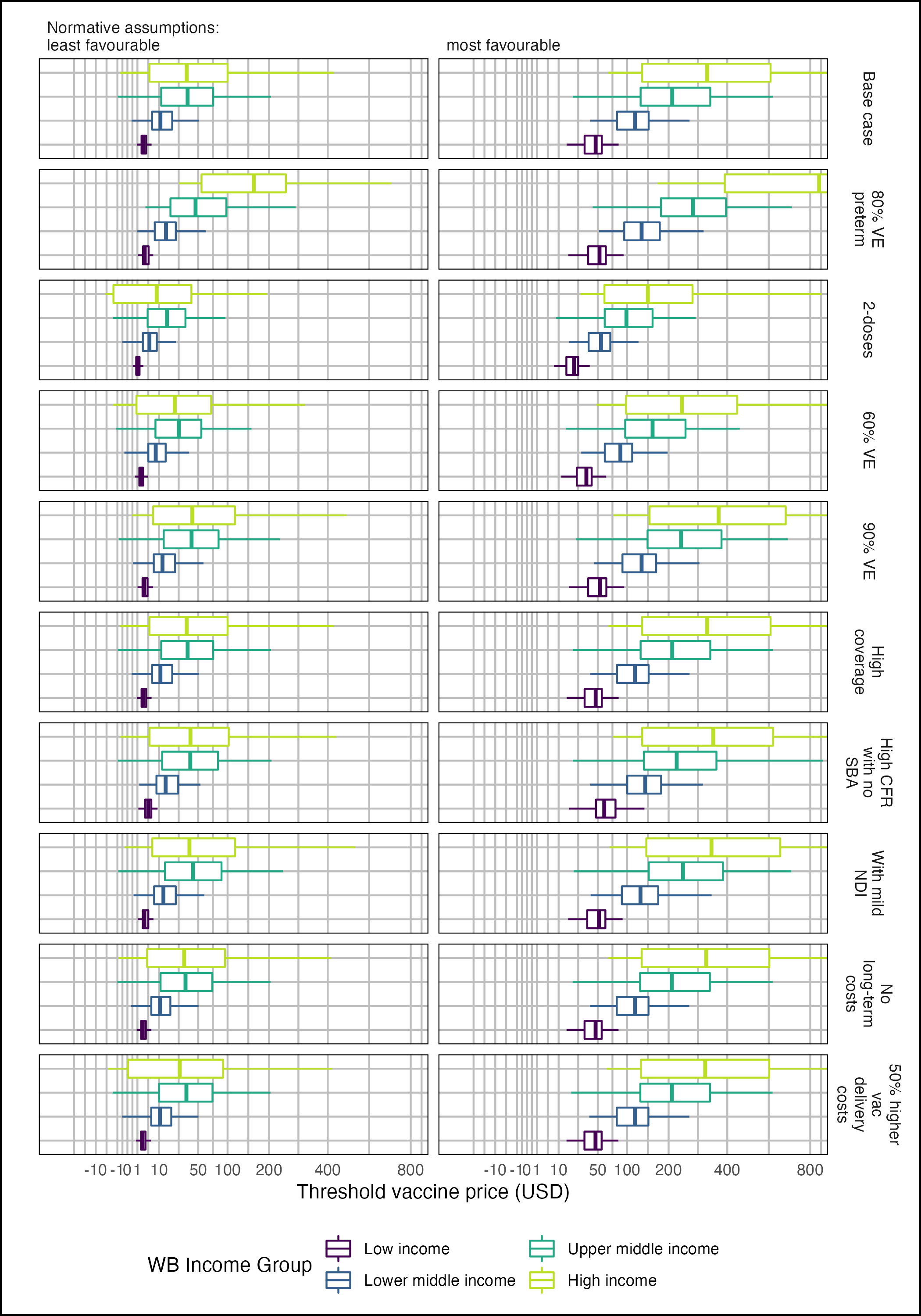
 Fig F:* Distribution of GBS vaccine threshold prices amongst countries within each World Bank region under most and least favourable normative assumptions for different vaccination scenarios.** Threshold vaccine prices above $800 per dose are not shown. Least-favourable normative assumptions were the use of an empirical CET, 3% discounting of QALYs, and exclusion of stillbirth QALYs. Most-favourable assumptions were the use of 1 x GDP per capita CETs, 0% discounting of QALYs, and inclusion of stillbirth QALYs. B = Billions; CET = Cost-Effectiveness Threshold; CFR = Case Fatality Risk; GDP = Gross Domestic Product; NDI = Neurodevelopmental Impairment; SBA = Skilled Birth Attendant; QALY = Quality Adjusted Life Year; VE = Vaccine Effectiveness

***Fig G:* Tornado diagram showing impact of varying individual model parameters on the estimated global Net Monetary Benefit using the least favourable normative assumptions.** Only the top 100 most influential parameters are shown. The impact of varying each parameter value between the 2.5% and 97.5% quantiles (shown by the different colour bars) was estimated using multi-variables linear regression. Regional parameters are shown by the region name in parentheses, country-level parameters by a suffix with the country iso3 code, and all other parameters are global. For the regression the risk of maternal colonisation and risk of EOGBS given colonisation were combined into a single risk of EOGBS parameter for each country to preserve the correlation between these two jointly sampled parameters. Least-favourable normative assumptions were the use of an empirical CET, 3% discounting of QALYs, and exclusion of stillbirth QALYs. CET = Cost-Effectiveness Threshold; EOGBS = Early-Onset-GBS disease; LOGBS = Late-Onset GBS disease; NDI = Neurodevelopmental Impairment

## A4 References

1. Husereau D, Drummond M, Petrou S, Carswell C, Moher D, Greenberg D, et al. Consolidated Health Economic Evaluation Reporting Standards (CHEERS) statement. BMJ. 2013;346: f1049–f1049. doi:10.1136/bmj.f1049

2. World Health Organization. WHO Preferred Product Characteristics for Group B Streptococcus Vaccines. Geneva; 2017.

3. World Health Organization. Global Health Observatory. [cited 2 Mar 2022]. Available: https://www.who.int/data/gho/

4. Malvolti S, Pecenka C, Mantel CF, Malhame M, Lambach P. A Financial and Global Demand Analysis to Inform Decisions for Funding and Clinical Development of Group B *Streptococcus* Vaccines for Pregnant Women. Clin Infect Dis. 2022;74: S70–S79. doi:10.1093/cid/ciab782

5. Procter SR, Salman O, Pecenka C, Gonçalves BP, Paul P, Hutubessy R, et al. A review of the costs of delivering maternal immunisation during pregnancy. Vaccine. 2020;38: 6199–6204. doi:10.1016/j.vaccine.2020.07.050

6. Salman O, Procter SR, McGregor C, Paul P, Hutubessy R, Lawn JE, et al. Systematic Review on the Acute Cost-of-illness of Sepsis and Meningitis in Neonates and Infants. Pediatr Infect Dis J. 2020;39: 35–40. doi:10.1097/INF.0000000000002500

7. Schroeder E-A, Petrou S, Balfour G, Edamma O, Heath PT. The economic costs of Group B Streptococcus (GBS) disease: prospective cohort study of infants with GBS disease in England. Eur J Health Econ. 2009;10: 275–285. doi:10.1007/s10198-008-0131-4

8. Aerts C, Leahy S, Mucasse H, Lala S, Bramugy J, Tann CJ, et al. Quantifying the Acute Care Costs of Neonatal Bacterial Sepsis and Meningitis in Mozambique and South Africa. Clin Infect Dis. 2022;74: S64–S69. doi:10.1093/cid/ciab815

9. Petrou S, Johnson S, Wolke D, Marlow N. The association between neurodevelopmental disability and economic outcomes during mid-childhood: Neurodevelopmental disability and economic outcomes. Child Care Health Dev. 2013;39: 345–357. doi:10.1111/j.1365-2214.2012.01368.x

10. Khavjou OA, Anderson WL, Honeycutt AA, Bates LG, Razzaghi H, Hollis ND, et al. National Health Care Expenditures Associated With Disability. Med Care. 2020;58: 826–832. doi:10.1097/MLR.0000000000001371

11. Petrou S, Krabuanrat N, Khan K. Preference-Based Health-Related Quality of Life Outcomes Associated with Preterm Birth: A Systematic Review and Meta-analysis. PharmacoEconomics. 2020;38: 357–373. doi:10.1007/s40273-019-00865-7

12. Bennett JE, Sumner W, Downs SM, Jaffe DM. Parents’ utilities for outcomes of occult bacteremia. Arch Pediatr Adolesc Med. 2000;154: 43–48.

13. Gonçalves BP, Procter SR, Paul P, Chandna J, Lewin A, Seedat F, et al. Group B streptococcus infection during pregnancy and infancy: estimates of regional and global burden. Lancet Glob Health. 2022; S2214109X22000936. doi:10.1016/S2214-109X(22)00093-6

14. Horváth-Puhó E, van Kassel MN, Gonçalves BP, de Gier B, Procter SR, Paul P, et al. Mortality, neurodevelopmental impairments, and economic outcomes after invasive group B streptococcal disease in early infancy in Denmark and the Netherlands: a national matched cohort study. Lancet Child Adolesc Health. 2021;5: 398–407. doi:10.1016/S2352-4642(21)00022-5

15. United Nations, Department of Economic and Social Affairs, Population Division. World Population Prospects 2019. Available: https://population.un.org/wpp/Download/Standard/Population/

16. Chawanpaiboon S, Vogel JP, Moller A-B, Lumbiganon P, Petzold M, Hogan D, et al. Global, regional, and national estimates of levels of preterm birth in 2014: a systematic review and modelling analysis. Lancet Glob Health. 2019;7: e37–e46. doi:10.1016/S2214-109X(18)30451-0

17. UNICEF. Delivery care. [cited 24 May 2022]. Available: https://data.unicef.org/topic/maternal-health/delivery-care/

18. The World Bank. GDP per capita (current US$). [cited 24 May 2022]. Available: https://data.worldbank.org/indicator/NY.GDP.PCAP.CD

19. International Monetary Fund. World Economic Outlook (April 2022) - GDP per capita, current prices. [cited 24 May 2022]. Available: https://www.imf.org/external/datamapper/NGDPDPC@WEO

20. Woods B, Revill P, Sculpher M, Claxton K. Country-Level Cost-Effectiveness Thresholds: Initial Estimates and the Need for Further Research. Value Health. 2016;19: 929–935. doi:10.1016/j.jval.2016.02.017

21. Ochalek J, Lomas J, Claxton K. Estimating health opportunity costs in low-income and middle-income countries: a novel approach and evidence from cross-country data. BMJ Glob Health. 2018;3: e000964. doi:10.1136/bmjgh-2018-000964

22. Blencowe H, Cousens S, Oestergaard MZ, Chou D, Moller A-B, Narwal R, et al. National, regional, and worldwide estimates of preterm birth rates in the year 2010 with time trends since 1990 for selected countries: a systematic analysis and implications. The Lancet. 2012;379: 2162–2172. doi:10.1016/S0140-6736(12)60820-4

23. Baral R, Fleming J, Khan S, Higgins D, Hendrix N, Pecenka C. Inferring antenatal care visit timing in low- and middle-income countries: Methods to inform potential maternal vaccine coverage. Larson BA, editor. PLOS ONE. 2020;15: e0237718. doi:10.1371/journal.pone.0237718

24. The World Bank. GDP deflator (base year varies by country) | Data. [cited 3 Mar 2022]. Available: https://data.worldbank.org/indicator/NY.GDP.DEFL.ZS

25. The World Bank. Official exchange rate (LCU per US$, period average) | Data. [cited 3 Mar 2022]. Available: https://data.worldbank.org/indicator/PA.NUS.FCRF
