# Supplementary material for: Maternal immunisation against Group B Streptococcus: A global analysis of health impact and cost-effectiveness
Source: PLoS Med. 2023 Mar 14;20(3):e1004068. doi: 10.1371/journal.pmed.1004068 (PMC10013922; doi:10.1371/journal.pmed.1004068)
Supplement: Attachment — Submitted filename: GBS CEA Response to reviewers letter_FINAL.docx [file pmed.1004068.s002.docx]

Dear Editor,

We would like to thank the reviewers and the editorial team for the helpful comments and suggestions, and for their patience during the delay in submitting this revised manuscript. We have incorporated our responses to specific points raised below.

Your sincerely,

Simon Procter (on behalf of all co-authors)

**Requests from the editors**:

Apologies for the delay in returning the manuscript to you. It took a long time to get reviewed, largely as result of multiple COIs. In addition, we are also unexpectedly extremely short staffed at the moment. The manuscript was reviewed by a subject reviewer, a methodological reviewer and an academic editor with relevant expertise. Their comments are included below.

GENERAL

Please address all editor and reviewer comments below.

Thank you for reporting your study according to the CHEERS 2022 statement. Given the nature of your study, it might be worth considering also the GATHER statement, further guidance can be found here: http://gather-statement.org/

Response: We acknowledge the importance of GATHER statements, and believe this is most relevant to the estimation of the GBS health-burden rather than economic evaluation:

“***GATHER*** *is designed to guide the reporting of estimates of a subset of health indicators: indicators of health status, such as estimates of total and cause-specific mortality and incidence and prevalence of diseases and injuries; and indicators of some proximal health determinants, such as health behaviors and health exposures.*”

Indeed we included a GATHER statement alongside our previously published burden estimates, which directly informed the current economic analysis (https://doi.org/10.1016/S2214-109X(22)00093-6). We have clarified this in the third paragraph of the methods sections as follows:

“**This analysis is reported in accordance with the** Consolidated Health Economic Evaluation Reporting Standards (CHEERS) checklist (supplementary appendix 1) **and is informed by the burden estimates that were reported previously according to the GATHER statement.**”

If necessary, then we could reproduce the GATHER statement for the burden estimates within the supplementary appendix of this manuscript.

ABSTRACT

* Please structure your abstract using the PLOS Medicine headings (Background, Methods and Findings, Conclusions).

Response: We have done this.

* Please combine the Methods and Findings sections into one section, “Methods and findings”.

Response: We have done this.

Abstract Methods and Findings:

* Please ensure that all numbers presented in the abstract are present and identical to numbers presented in the main manuscript text.

Response: Thank you for noticing this discrepancy, we have now amended the abstract so that the figures match those in the main text.

* Please expand the description of the study design which is rather brief – size of female cohort? any important dependent variables that are adjusted for/considered?

Response: We have added some additional details on the size of the cohort of pregnant women, and the calculation of costs and QALYs.

* In the last sentence of the Abstract Methods and Findings section, please describe the main limitation(s) of the study's methodology.

Response: We have added a sentence on the main study limitation.

Abstract Conclusion

Please remove the heading “interpretation” and instead include a conclusions section considering the following points:

* Please address the study implications without overreaching what can be concluded from the data; the phrase "In this study, we observed ..." may be useful.

* Please interpret the study based on the results presented in the abstract, emphasizing what is new without overstating your conclusions.

* Please avoid vague statements such as "these results have major implications for policy/clinical care". Mention only specific implications substantiated by the results.

* Please avoid any assertions of primacy ("We report for the first time....")

Response: We have replaced the “interpretation” section with a “conclusions” section. Having added the additional details requested to the “Methods and Findings” section the abstract is already quite long (375) so we have deliberately kept the conclusions short and focused only on the global results presented in the abstract.

AUTHOR SUMMARY

At this stage, we ask that you include a short, non-technical Author Summary of your research to make findings accessible to a wide audience that includes both scientists and non-scientists. The Author Summary should immediately follow the Abstract in your revised manuscript. This text is subject to editorial change and should be distinct from the scientific abstract. Please see our author guidelines for more information: https://journals.plos.org/plosmedicine/s/revising-your-manuscript#loc-author-summary

Response: We have added an author summary, which is reproduce below for convenience:

“*Why was this study done?*

Group B Streprococcus (GBS) is a common bacterial pathogen that can infect pregnant women and their babies.

- A recent global disease burden study showed that GBS infection causes a considerable burden of sepsis and meningitis in newborns which can sometimes result in death or long-term disability, and it may also be linked to increased risk of stillbirth and preterm births.
- Several vaccines against GBS for use during pregnancy are being developed.
- A global economic evaluation of GBS vaccines is needed to inform investment decisions in vaccine development and to guide fair financing and pricing to enable equitable access once licensed vaccines become available.

*What did the researchers do and find?*

- We developed a decision model to assess the cost-effectiveness of GBS vaccines in pregnant women in 183 countries for the year 2020.
- Our model used the most recent global estimates of the health burden of GBS in pregnant women and their children together with estimated costs to healthcare systems.
- We found that globally a maternal GBS vaccination programme, integrated in antenatal care, would lead to an overall increase in costs that are partially offset by savings in healthcare costs, along with substantial health gains notably reductions in morbidity and mortality.
- Globally, the value of the annual GBS vaccine programme ranged from $1.1 billion (95% uncertainty range: $-0.2 – 3.8 billion) to $17 billion ($9.1 – 31 billion) depending on the normative assumptions used by policymakers.

*What do these findings mean?*

- Globally, GBS maternal immunisation is likely to be cost-effective and avert a substantial burden of death and disability in children.
- At a regional and county level cost-effectiveness is sensitive to vaccine prices and to different choices policymakers may use to value benefits in improved health.
- Our findings highlight the need both for carefully tiered vaccine pricing to ensure equitable access across countries, and for local assessment of cost-effectiveness as GBS vaccine moves towards licensure.
- There is a need for more evidence on the impact of GBS on several outcomes, including stillbirths, preterm births, and maternal morbidity, as well as the wider societal costs of long-term GBS-related disability.”

METHODS and RESULTS

Ordinarily we would ask that main results are quantified with 95% CIs and p-values (both in the abstract and in the main manuscript). I understand the use of uncertainty intervals/ranges in a study of this kind. Where possible and appropriate, please also include p-values, if not please give a brief justification. If/where p-values are presented please also provide the statistical test used to determine them.

Response: Thank you, 95% CIs are applicable to frequentist statistical analysis, and it is not possible to present either 95% CIs or p-values for our analysis, which uses a Bayesian framework. There is no formal hypothesis being tested, and the 95% uncertainty intervals presented should be interpreted as a range covering 95% of the probability density of the estimated outcome conditioned on the data and the modelling assumptions. Note that presentation of uncertainty intervals only is the standard practice in studies that report population-level burden and cost estimates (Claxton <https://doi.org/10.1016/S0167-6296(98)00039-3>).

Line 107 “We developed a decision-tree model (Fig. 1)” please revise to “Figure. 1” Please check and amend throughout the manuscript.

Response: We have replaced all instances of “Fig.” with “Figure”.

Line 109 “The size of the cohort of women in each country was calculated by…”. It's not immediately obvious what the cohort size was, it takes a bit of looking and reading of different parts to appreciate this. Suggest revising for improved clarity/reader accessibility

Response: Thank you for highlighting this, which was confusingly written. We have added the total size of the cohort of pregnant women across included countries (140 million) and clarified that this is based on the UNWPP estimates of the number of births. This relevant part of this paragraph now reads as follows.

“We developed a decision-tree model (Figure. 1) to assess the health impact and cost-effectiveness of maternal vaccination against GBS in an annual cohort of 140 million pregnant women and their babies for the year 2020 compared with current practice of no vaccination. The size of the cohort of pregnant women in each country was based on country-specific estimates of the number of births from the United Nations World Populations Prospects (UNWPP).”

Line 267 please remove the funding statement from this section and include in the manuscript submission form

Response: We have removed this from the manuscript.

FIGURES

These are very nicely presented and well described. Throughout including supplementary files, please consider making them accessible to those with colour blindness (i.e. avoiding the use of red/green)

Response: Thank you for this suggestion to help improve the accessibility of our manuscript. We have reproduced the figures using the Viridis colour-blind friendly colour palette.

TABLES

Where appropriate please also include p-values (as above)

Response: Please see response above about why presenting p-values is not appropriate for this analysis.

DISCUSSION

Please ensure the Discussion is presented as follows: a short, clear summary of the article's findings; what the study adds to existing research and where and why the results may differ from previous research; strengths and limitations of the study; implications and next steps for research, clinical practice, and/or public policy; one-paragraph conclusion.

Response: We think the discussion already follows this format, but would be happy to give consideration to any specific guidance on elements that require changing.

Line 435 – please remove the declaration of interests statement and include only in the submission form

Response: We have removed this from the manuscript.

REFERENCES

Please select the PLOS Medicine reference style in your citation manager. For in-text reference call outs, citations should be in square brackets, and preceding punctuation, note the absence of spaces within the square brackets, “…symptomatic [2,8].”

In the bibliography please ensure no more than 6 authors are listed before et al where more than n=6 authors contribute. Journal name abbreviations should be those found in the National Center for Biotechnology Information (NCBI) databases.

Response: We have amended the citation and bibliography styles as requested.

**Comments from the academic editor**:

I have now read the reviewers' comments and the submitted paper and agree with the suggestion of (to me) major changes. Reviewer 3 in particular gives comments that would require some additional analysis which is entirely doable.

It is an interesting paper focussing on an important public health infection - although given that no vaccine is currently available this is a bit hypothetical but could push the vaccine development agenda.

I look forward to seeing the revised manuscript.

Response: Thank you. We have responded to the specific comments from the reviewers below.

Comments from the reviewers:

**Reviewer #1**: This is a well-conducted global cost-effectiveness analysis on the impact of maternal immunisation against Group B Streptococcus. The study design, datasets, statistical methods and analyses, and presentation (tables and figures) and interpretation of the results are mostly adequate and of a very good standard. However, there are still a few minor points needing attention.

Response: Thank you.

1) One of the key findings is "Estimated global NMB ranged from $1.1 billion ($-0.2 - 3.8 billion) to $17 billion ($9.1 - 31 billion)", which came from the results in Figure 4. The readers could try their best to find this conclusion from Figure 4, however as this is such an important result, it would be good for authors make it absolutely clear and highlighted in the main text on exactly where this lower limit (1.1 billion) and higher limit (17 billion) came from and under what assumption and scenario. Also need to mention, the 95% CI for the lower bound estimate includes 0, which strictly speaking is not statistically significant - meaning under least favourable assumption/senario the proposed vaccine may not be cost-effective, which needs to be discussed in balance with the positive results under the favourable situation.

Response: Thank you, we have included an extra sentence (see below) mentioning that the uncertainty range for the least favourable assumptions includes zero as we agree this is important to highlight. We note that for a cost-effectiveness analysis conventional levels of statistical significance are not necessarily appropriate, since the aim is not to have a certain level of confidence about a hypothesis, but to select an option from alternatives that maximises efficiency, based on the expected value of the decision. (Claxton <https://doi.org/10.1016/S0167-6296(98)00039-3>).

For the NMB range quoted in the main text we refer the reader to Figure 2A (reproduced below for convenience) which shows the NMB under various different normative assumptions (although the lower/upper values are also presented in the base case in Figure 4) and have also referred to reader to Table 1 for definitions of the different normative assumptions. We have adjusted the main text to make clear that the lower and higher limits of the 1.1 to 17 billion range correspond respectively to the least/most favourable assumptions.

“Using our base case assumptions about the vaccine characteristics the estimated global NMB of vaccination ranged from $1.1 billion (UR: $-0.2 – 3.9 billion) under least favourable normative assumptions to $17 billion (UR: $9.1 – 31 billion) under most favourable depending upon the normative assumptions (Table 1 and Figure. 2A). Including stillbirth QALYs increases the NMB by between $1.4 billion and $7.1 billion depending on the other normative assumptions made. Although the point estimate was positive under all assumptions, for the least favourable assumptions the uncertainty range includes zero.”

2) Figure 4. Under the least favourable assumption, when looking at the 95% CI of many scenarios, the NMBs are not statistically significant (being positive) such as for 60% VE, 2 dose, high coverage and base case, which means the vaccine may not be cost-effective under these circumstances. I can see the authors have discussed the uncertainties in the discussion, however it would be good to go a bit further on how to make an effective implementation of the proposed vaccine program to avoid these pitfalls given that the sensitivity/scenario analyses have already usefully identified those problems.

Response: As noted above, meeting conventional statistical significance is not generally the main consideration for a CEA - in order to maximise economic benefit decision makers should focus on the expected net benefit. For most of the scenarios the majority of the probability is above zero suggesting that globally the NMB is likely positive. The global uncertainty ranges are partly the result of regional/country heterogeneity (supplementary figures 2 and 4). In some scenarios (such as 60% VE and two-dose vaccination) there are many countries where vaccination is likely not cost-effective under least favourable normative assumptions. This information is relevant for vaccine developers trying to formulate vaccines that will deliver net benefits. From an implementation perspective we believe the key is to obtain vaccines at the best possible price and to undertake analysis tailored to the country context as new data on key vaccine parameters becomes available.

We have added the following sentence to the penultimate paragraph of the discussion:

“As GBS vaccines progress towards licensure, and new data on vaccine characteristics and GBS epidemiology become available, updated analyses tailored to individual country contexts can help ensure that vaccines are acquired at prices that are cost-effective.”

**Reviewer #2**: Thank you for your revised manuscript. I have no further comments and am very much looking forward to seeing this in print

Response: Thank you.

**Reviewer #3**: In their analysis, Procter and co-authors conducted a model-based cost-effectiveness analysis to project the health and economic consequences of a hypothetical global Group B Streptococcus maternal immunization program. This analysis was the first to incorporate the new GBD projections and synthesize outcome across 170+ countries. The analysis and manuscript are generally comprehensive, transparent and well-executed. I only have minor comments and clarifying questions.

Response: Thank you.

1. Abstract. Page 2, line 45-46, could the authors concisely describe what this range reflects?

Response: This range reflects the least/most favourable normative assumptions used in the valuation to calculate the NMB. We have amended the sentence as follows:

“Estimated global NMB ranged from $1.1 billion ($-0.2 – 3.8 billion) **under the least favourable normative assumptions** to $17 billion ($9.1 – 31 billion) **under the most favourable normative assumptions**.”

2. Page 6, line 171. I apologize if I missed it, but do you describe the utility decrements/QALY loss assumptions for newborn iGBS death? It is mentioned briefly later on (page 8), but could the authors describe how it's calculated, and the value?

Response: An iGBS death is assigned zero QALYs, i.e. an iGBS death results in QALY loss equivalent to average life expectancy. We have amended the first sentence in this paragraph to clarify this:

“To calculate QALYs we assumed country-specific life-expectancy at birth for both normal births and survivors of iGBS, **and assigned zero QALYs for an iGBS death**.”

3. Page 7, line 179. For survivors with long-term sequelae, did the authors need to assume an average age for the onset of LOGBS to determine at what age to start applying utility decrements? Similarly, for EOGBS?

Response: We have clarified that, since invasive GBS disease cases occur in the first months of life, we assumed this utility decrement to apply from birth (for both EOGBS and LOGBS). This is because the extra few weeks we may have added to the duration by assuming an earlier point of disease are insignificant compared to the decades of life that the sequelae can last.

4. Page 8, line 217. Small typo at the end of the line.

Response: Thank you, we have corrected this.

5. Page 9, line 261. Could the authors briefly describe their approach to assigning distributions for the reader. In the supplementary appendix table 2, I noticed several risk parameters were assumed to be fixed, e.g., Riskmild-NDI-men. Could the authors justify? Was any correlation between parameters assumed? Is the 2.5 and 97.5 considered across all countries as well as across simulations?

Response: We have tried to provide more clarity regarding the choice of parameter distributions and have amended the final paragraph (see below.)

“To account for combined parameter uncertainty, for each scenario we ran 4000 simulations per country sampling parameters from their corresponding probability distributions (supplementary table 4) and calculated the median and 95% uncertainty range (UR) based on 2.5 and 97.5 percentiles of the simulations. For costs we used log-normal distributions, for health-state utility values we used beta distributions, and for epidemiological parameters we sampled from the posterior distributions from the Bayesian burden model. Due to lack of data, we assumed fixed values for the background risks of NDI after sepsis and meningitis not due to iGBS in line with the approach used in the global burden analysis. We also assumed fixed values for vaccine characteristics, since these are currently unknown and we instead varied these in scenario analysis. Similarly, we used fixed vaccine prices, but as a sensitivity analysis estimated the threshold price at which a GBS vaccine would be cost-effective in each country. At the country level we used the simulation results to calculate the probability maternal GBS vaccination was cost-effective (i.e., the proportion of simulations with NMB > 0) under different scenarios.”

On the second point the risk parameters that are assumed to be fixed are the baseline risk of mild NDI and the risk of moderate/severe NDI without iGBS, in the base-case analysis we additionally assume that the risk of mild NDI after GBS sepsis or meningitis is fixed at the same value as the baseline risk of mild NDI. This has the effect of conservatively assuming that there is no excess risk of mild NDI due to iGBS (footnote g of Supplementary Table 2). There are a few reasons why we did not assume a distribution for the baseline risks of NDI:

- To be consistent with the approach and values used for moderate/severe NDI in the previous global burden of GBS study (Goncalves et al. <https://doi.org/10.1016/S2214-109X(22)00093-6>). The data used to inform the estimation of the baseline risk in that study were from a large cohort, with a large sample size, implying that the uncertainty for that population was limited, and to our knowledge the only analysis describing the NDI risk in groups known to not have a history of iGBS. However, it is possible that the value of this parameter varies by region or country, but this has not been quantified.
- The uncertainty in the posterior risk of NDI after sepsis/meningitis is already very wide, and the baseline risk of NDI and the risk of NDI after iGBS are likely to be highly correlated. We therefore chose to make a simple assumption of fixed baseline risk, which we have now made clearer in the text.
- To be conservative in our base case analysis we assumed that the excess risk of mild NDI due to GBS was zero.

We additionally, note that previous cost-effectiveness analyses of GBS vaccines have generally assumed that the risk of NDI following iGBS is all due to GBS, so our inclusion of the baseline risk although crude is more conservative than previous analyses.

6. Methods. A Bayesian disease model is mentioned in the abstract methods but is not mentioned in the main manuscript until the discussion section. Consider describing this model in some detail within the main manuscript methods.

Response: Thank you, we now realise that this may have been somewhat unclear and have modified the first sentence of the second paragraph in the methods to read as follows.

“The health impact model structure was designed to reflect the natural history of pregnancy related GBS infections and was aligned with the **Bayesian disease** modelling framework used in recently reported global estimates of GBS burden. **The Bayesian disease model has been described in detail elsewhere and its structure is reflected in our methods below.**[1,26]

We hope this will make the link to the previously published Bayesian disease burden model clear (<https://doi.org/10.1016/S2214-109X(22)00093-6>). However, we do not have the space to describe this model in any more detail in the current manuscript, and prefer to refer the reader to the previous publication where this model is summarised in the methods of the main manuscript and described in detail in the supplementary appendices.

7. Results. Although the authors transparently report the distributions and parameterization of their uncertainty, I am left wondering what parameter(s) is(are) driving the large uncertainty bounds in the outcomes. If the bounds included uncertainty across the countries, this between-country variation may be a driver (see comment 5)). It would be informative for the reader if a regression or EVPPI analysis could be undertaken to shed light on the drivers of the uncertainty points for at least the main outcome of NMB; although, such analysis would only point to parameters that were not fixed (and descriptions would need to be set in the context of fixed vaccine efficacy).

Response: Thank you for this comment. We agree that it would be helpful to provide more insight into which are the most important parameters contributing to the uncertainty in the estimates. As suggested, we have undertaken a multi-variable regression analysis to highlight the most influential parameters on global NMB, and have presented this as a tornado diagram in the supplementary material (and reproduced below for convenience.)

We add the following to the final paragraph of the methods:

“We used multivariate linear regression to assess the influence of individual model parameters on the uncertainty of our estimates of global NMB.”

We added the following to the penultimate paragraph of the results section:

“Supplementary Figure 7 shows the model input parameters that had the greatest influence on the uncertainty of the NMB estimates under least favourable normative assumption. Globally, NMB was most sensitive to variation in the risk of EOGBS (particularly in countries with higher numbers of births and higher GDPPC, e.g. China and USA), the proportion of iGBS that is late-onset, Case Fatality Risk, and the risk of developing moderate/severe NDI.”

8. Page 13, line 319. To categorize the 103 countries, do the authors assumed a cut-point of 50% probability of being cost-effective, or are the authors using expected NMB >0 for the cutoff (preferred)?

In general, I would challenge the co-authors about what additional information fig 3 adds to the messaging in order to justify inclusion, given that readers often mis-interpret the probability of an intervention being cost-effective. Although this figure reports individual-country uncertainty, the expected NMB with uncertainty bounds are reported nicely in Figure 2 (presenting the expNMB, which are used to make decisions), and I wonder if the same approach could be used for the individual-level country's NMB?

Response:

Upon reflection, we agree with the reviewer. We had categorised country-level cost-effectiveness when based on the probability of being cost-effective over 50% but have now changed this to use expected NMB above zero. We have clarified this in the text and updated the figures in the text which slightly changed.

“For the most-favourable normative assumptions vaccination is likely cost-effective **(mean NMB > 0)** in almost all countries (Figure 3.), but for least-favourable assumptions this was reduced to **around 60%** (**112**/183) of countries.”

Our main argument for including figure 3 is to highlight that within regions there is between country heterogeneity of cost-effectiveness (under less favourable normative assumptions) We did look at alternative presentations of this data, and the reason for presenting the probability of cost-effectiveness rather than expected NMB is to allow the data for countries with numerically very different NMB values to be presented on the same y-axis. While, perhaps not as directly interpretable as presenting NMB directly, this presentation still conveys something about the uncertainty in each country under the different scenarios (i.e. p(cost-effective) = 1 implies lower bound of NMB > 0, while p(cost-effective) = 0 implies upper bound of NMB < 0).
