# Supplementary material for: Maternal immunisation against Group B Streptococcus: A global analysis of health impact and cost-effectiveness
Source: PLoS Med. 2023 Mar 14;20(3):e1004068. doi: 10.1371/journal.pmed.1004068 (PMC10013922; doi:10.1371/journal.pmed.1004068)
Supplement: Attachment — Submitted filename: GBS CEA Response minor revisions_FINAL.docx [file pmed.1004068.s003.docx]

Dear Editor,

We are pleased to submit the minor revisions to our manuscript. Specific changes in responses to the points raised are noted below.

Your sincerely,

Simon Procter (on behalf of all co-authors)

Requests from Editors:

GENERAL
Thank you for your detailed and considerate responses to previous editor and reviewer comments which we appreciate and accept. Please see below for some further minor revisions.

Thank you, we have addressed the minor revisions.

FIGURES
Thank you for revising the colour palate used for your figures. Figure 1 remains unchanged, at least in my version, please revise if possible.
Previously I said the following: “Line 107 We developed a decision-tree model (Fig. 1) - please revise to “Figure. 1” Please check and amend throughout the manuscript. This was my error, please accept my sincere apologies. Figures should be labelled as “Fig” and referred to in the text as such.

We have amended to “Fig” throughout the manuscript and supplementary appendix.

DISCUSSION
Line 446: “Our study is the first…” claims of primacy can be risky, suggest “To our knowledge…” or something similar

Thank you, we have amended as suggested.

REFERENCES
For in-text reference callouts, please ensure that citations (placed in square parentheses) precede punctuation. For example, line 448 should read “…United States [12–14], the Netherlands [17],” and line 449 should read “…37 Gavi countries in Africa [20].” 
Please check and amend throughout

We have done this.

SOCIAL MEDIA
To help us extend the reach of your research, please provide any Twitter handle(s) that would be appropriate to tag, including your own, your coauthors’, your institution, funder, or lab. Please detail any handles you wish to be included when we tweet this paper, in the manuscript submission form when you re-submit the manuscript.


Comments from Reviewers:

Reviewer #1: Thanks authors for their effort to improve the manuscript. I am satisfied with the response and revision. No further issues needing attention.

Thank you.

Reviewer #3: Thank you for your thoughtful responses. I have no further comments.

Thank you.
